# Prevalence, predictors, and clinical relevance of drug–drug interactions in outpatient prescribing: A national cross-sectional study

**DOI:** 10.1371/journal.pone.0345076

**Published:** 2026-04-08

**Authors:** Seyed Sahab Aarabi, Farbod Semnani, Zahra Aminzade, Reza Mehrizi, Mohammad Gholamnezhad, Raham Armand, Nezam Armand, Leila Ghamkhar

**Affiliations:** 1 National Center for Health Insurance Research, Tehran, Iran; 2 School of Medicine, Tehran University of Medical Sciences (TUMS), Tehran, Iran; 3 School of Medicine, Shahid Beheshti University of Medical Sciences (SBMU), Tehran, Iran; 4 Endometriosis Research Center, Iran University of Medical Sciences (IUMS), Tehran, Iran; 5 Clinical Research Development Unit, Yasuj University of Medical Sciences, Yasuj, Iran; 6 Behbahan Faculty of Medical Sciences, Behbahan, Iran; 7 Department of Health Management, Policy, and Economics, School of Public Health, Tehran University of Medical Sciences, Tehran, Iran; University of Science and Technology of Fujairah, YEMEN

## Abstract

**Background:**

Drug–drug interactions (DDIs) represent a major preventable cause of medication-related harm globally. Their prevalence varies across health systems, but common drivers include polypharmacy, aging populations, and specialty-specific prescribing patterns. Large-scale pharmacoepidemiologic analyses of real-world prescription data can clarify the magnitude of the problem and inform strategies to reduce risks.

**Methods:**

This retrospective study included 2,365,811 outpatient prescriptions (982,102 patients) from Tehran, Iran. The top 100 most prescribed medications were screened for potential DDIs via Micromedex®. Interactions were classified as contraindicated, major, or moderate. Logistic regression identified demographic, specialty, and prescription-level predictors.

**Results:**

Potential DDIs occurred in 46.1% of prescriptions, with 57.8% of patients affected. Major DDIs (62.6%) dominated, followed by moderate (32.8%) and contraindicated (4.6%). ASA was a frequent contributor to high-risk pairs. Contraindicated interactions were largely NSAID duplications, most common in orthopedics and emergency medicine. Psychiatry and cardiology prescriptions showed the highest prevalence, while polypharmacy strongly predicted DDIs, with incremental risks amplified in older adults.

**Conclusions:**

Outpatient DDIs represent a substantial burden in routine care, comparable to international reports. Prevention requires comprehensive strategies, including e-prescribing with CDSS, pharmacist-led reviews, patient education on OTC use, and policy interventions to limit reimbursement of unsafe combinations.

## Introduction

Drug–drug interactions (DDIs) pose a major challenge in healthcare, resulting from one medication altering the pharmacological effect of another drug that is given at the same time [1]. A High prevalence of the DDI has been reported across different populations, with recent analyses showing rates of 12.12% and 10.06% in Indianapolis (USA) and Catalonia (Spain), respectively [2]. In low and middle-income countries (LMICs), the situation is even more concerning. This disparity is often attributed to limited resources for medication monitoring and insufficient clinical decision support systems in resource-constrained settings [3,4]. While several studies have explored DDIs within the Iranian healthcare context, their findings show significant variability. For instance, a systematic review of Iranian studies reported a median DDI rate of 8.5% in the outpatient setting. However, individual reports vary widely. This wide disparity is largely related to the small or single-center sample size and the lack of a unified approach [5]. There remains a critical need for large-scale, methodologically standardized pharmacoepidemiologic analyses to bridge these gaps and provide a baseline for national health policy reforms.

The clinical consequences of DDIs vary from minimal effects to significant adverse events that may include life-threatening ones [6]. Exposure to DDI is associated with an increased number of adverse drug events and decreased health‐related quality of life [7]. Pharmacokinetic interactions can result in altered drug absorption, distribution, metabolism, or excretion, while pharmacodynamic interactions may produce additive, synergistic, or antagonistic effects [8]. Severe DDIs may present as cardiac arrhythmias, seizures, respiratory depression, hypotension, kidney injury, liver toxicity, or bleeding [9]. DDIs could be responsible for a large number of hospital admissions, with anticoagulants, cardiovascular drugs, and antimicrobials commonly involved [7,10]. In addition to physiological damage, DDIs could have a significant impact on treatment adherence since patients suffering from adverse effects could stop taking life-saving medications without consulting their medical doctor [11].

DDIs also represent a critical threat to patient safety within polypharmacy, defined as concurrent use of five or more medications [12]. Likewise, such a threat exists regarding multimorbidity, where complex regimens amplify DDI risk, adverse drug reactions, and hospitalizations, particularly in older adults. Patient safety frameworks, such as WHO’s Medication Without Harm initiative, emphasize clinical decision support systems and deprescribing protocols to mitigate these risks, emphasizing the need for tailored pharmacoepidemiologic strategies in at-risk populations [13].

Discovery and management of DDIs can substantially decrease healthcare utilization and costs [7]. This economic impact is particularly pronounced in healthcare systems with limited resources, where preventable DDIs divert scarce funding from other essential services [14].

Several tools are available to healthcare providers for the timely identification of potential DDIs, as early interventions prevent malefic outcomes [15]. The Micromedex Drug Interactions database is one of the most commonly used and extensive resources available, providing evidence-based interventions to support clinical decisions across the patient care continuum [16]. Screening of DDIs confirmed that although newer solutions are promising, established databases like Micromedex remained unrivaled in accuracy, breadth of coverage, and showed the best performance [15,17].

Therefore, this study aimed to evaluate outpatient medication safety in Iran by achieving three primary objectives: (1) determining the prevalence and patterns of potential DDIs at patient and prescription levels; (2) classifying these interactions by severity patterns. These include contraindicated, major, and moderate interactions based on Micromedex® criteria (a top clinical decision support package); and (3) identifying demographic and clinical predictors of DDI occurrence and severity. Characterizing DDIs at a national level provides the essential evidence required to refine clinical prescribing guidelines, optimize decision support within e-prescribing systems, and implement health policy reforms to improve medication safety.

## Materials & Methods

### Study design and data source

This was a retrospective, cross-sectional study that analyzed data from the Iran Health Insurance Organization (IHIO) database for the year 2022–2023. The primary objective was to investigate the prevalence and patterns of potential DDIs, categorize them by severity, and identify associated demographic and clinical predictors. The dataset was accessed for research purposes on February 19, 2025. All data were fully anonymized prior to analysis, and the authors had no access to any information that could identify individual participants during or after data collection. Therefore, in accordance with institutional and national ethical guidelines, formal ethics approval and informed consent were not required. This study adheres to the Strengthening the Reporting of Observational Studies in Epidemiology (STROBE) reporting guidelines for cross-sectional studies.

The study protocol was reviewed and conducted in accordance with the national ethical standards of the National Center for Health Insurance Research and the Iran Health Insurance Organization (IHIO) allowing the use of administrative health data. As this was a retrospective, cross-sectional analysis utilizing a fully de-identified national dataset, the research was classified as exempt from individual informed consent.

### Data processing and cohort selection

Data processing and cohort cleaning were conducted using Python software (Version 3.12). We included all IHIO-registered outpatient prescriptions in Tehran between March 26, 2022, and February 25, 2023. Prescriptions were excluded if they had incomplete information on patient sex or age, contained only a single drug, or did not include at least one drug from a list of the 100 most frequently prescribed medications in the database.

### Drug-drug interaction identification

A list of the 100 most frequently prescribed drugs was used as a reference. A comprehensive analysis was performed to identify all possible DDIs among these drugs using the Micromedex Drug Interactions database. Micromedex categorizes drug interaction severity into four levels: contraindicated, major, moderate, and minor. Contraindicated means the two drugs should not be taken together because the risk of serious harm is very high. Major interactions may be life-threatening and/or demand urgent medical management to prevent severe adverse outcomes. Moderate interactions may worsen the patient’s condition and/or necessitate adjustments in treatment [18]. The DDI information containing contraindicated, major, and moderate was first exported from Micromedex online database as PDF files for each of these 100 drugs, which were manually reviewed, cleaned, and semi-automatically converted to CSV files for use in the analysis. Minor interactions were excluded from the analysis because they are generally considered clinically negligible and typically do not necessitate treatment modifications. To ensure the consistency and accuracy of this process, two authors (S.S.A. and F.S.) independently performed the data extraction and cleaning. A cross-check of the converted CSV files against the original PDF records was conducted, and any inconsistencies in drug mapping or severity grading were resolved through consensus.

To accurately identify interactions, a multi-step process was implemented. Regular expressions were used to match generic drug names from the Micromedex data to the various brand names and formulations found in the IHIO prescription records [19]. For generic drugs like “Ferrous Sulfate,” a list of alternative names such as “IRONFORTE,” “FERFOLIC,” and “FOLIRON” was manually curated and used in a regex pattern to ensure all relevant prescriptions were captured. The analysis focused on systemic drugs, and non-systemic drugs were excluded by identifying and omitting prescriptions containing keywords like cream, lotion, and drops in the drug name. Additionally, to avoid double-counting, drug pairs were standardized by sorting the names alphabetically (e.g., Drug A-Drug B was treated the same as Drug B-Drug A). For prescriptions with multiple interactions of varying severity for the same drug pair, the highest severity level was retained for analysis. All Python scripts used for data processing and analysis are openly available in our GitHub repository (https://github.com/SahabAarabi/Drug-Drug-Interaction-Project).

### Statistical analysis

The prevalence of DDIs was calculated at both the patient and prescription levels. The frequency and proportion of DDIs by severity level were also analyzed. Based on potential violations of the normality assumption assessed using Shapiro-Wilk tests, data are reported as either mean ±SD or median and Inter-quartile range (IQR). Differences between groups were assessed using student's t-tests, analysis of variance (ANOVA), and chi-squared (χ²) tests. A p-value threshold of 0.05 was utilized for statistical significance.

To identify factors independently associated with the number and severity of DDIs, multivariable regression models were employed using R software (R Core Team (2024). R: A Language and Environment for Statistical Computing. R Foundation for Statistical Computing, Vienna, Austria. https://www.R-project.org/, version 4.5.0). R scripts used for analysis are also openly available in the mentioned GitHub repository.

A linear regression model was developed to predict the total count of DDIs per prescription. The model included independent variables such as age group, sex, drug count, and physician specialty. An additional model was constructed to explore the interaction effects between drug count and age group, as well as drug count and specialty, to determine how the effect of polypharmacy varies across different patient and physician groups. Moreover, a logistic regression model was utilized to predict the presence of a contraindicated DDI (a binary outcome of yes or no). The same set of independent variables from the linear model was used to identify key predictors of these high-risk interactions. Results are presented as odds ratios (OR) with 95% confidence intervals (CI) to quantify the magnitude of each predictor’s effect. Model robustness was evaluated using the adjusted R^2^ for linear regression models and the Akaike Information Criterion (AIC) for the logistic regression model. Multicollinearity was assessed for all multivariable models using the Variance Inflation Factor (VIF). All VIF values were found to be below 3, confirming that collinearity did not significantly bias the model estimates. Wherever model assumptions (e.g., normality, homoscedasticity, or linearity of predictors) were not fully met, we applied a nonparametric bootstrap approach with 1,000 resamples to obtain robust estimates and validate model stability.

A drug interaction network was created to visually represent the most common DDIs. This was accomplished using Cytoscape software [20]. In this network, drugs were represented as nodes, with the size of each node scaled to reflect its total interaction count. The connections between drugs (edges) were weighted by the frequency of co-prescription and colored according to the severity of the interaction (red for contraindicated, orange for major, and green for moderate).

## Results

From an initial 3,093,481 prescriptions (16,421,497 drugs), 727,670 were excluded. Specifically, 35,913 prescriptions were removed due to incomplete demographic data (missing age or sex), 128,638 were excluded because they contained only a single drug, and 563,119 were removed as they did not include any of the 100 most frequently prescribed medications. This resulted in 2,365,811 prescriptions comprising 11,499,301 drugs from 982,102 patients. The cohort had a median age of 51 years (IQR: [31–64]). On average, each patient received 2.41 ± 2.35 prescriptions, containing 4.86 ± 2.43 medications in each prescription. Nearly half of all prescriptions and more than 60% of patients involved polypharmacy (≥ 5 drugs), underscoring substantial exposure to potential drug interactions. Sex-specific patterns showed females had more prescriptions per patient, while males had more drugs per prescription and higher polypharmacy prevalence (p < 0.001). Furthermore, both prescription frequency and polypharmacy rates rose significantly with age. Patient characteristics are detailed in Table 1.

**Table 1:** Baseline characteristics of the selected cohort.

Variables		Patients N (%)	Age Median (IQR)	Prescription per patient (mean ±SD)	Medications per prescription (mean ±SD)	Patients with polypharmacy* (N, %)	Prescriptions with polypharmacy (N, %)
Sex	**Female**	569,185 (57.96)	**51 (34–63)**	**2.46 ± 2.34**	**4.85 ± 2.43**	**345,128 (60.63)**	**663,904 (47.32)**
	**Male**	412,917 (42.04)	**51 (26–64)**	**2.33 ± 2.36**	**4.87 ± 2.44**	**244,704 (59.26)**	**460,193 (47.80)**
Age group (years)	**0-18**	135,836 (13.84)	10 (6 - 14)	**1.86 ± 1.48**	**4.4 ± 2.0**	**66,907 (49.26)**	**98,265 (38.94)**
	**19-39**	194,263 (19.78)	30 (23 - 35)	**1.83 ± 1.6**	**4.78 ± 2.36**	**108,872 (56.04)**	**166,736 (46.67)**
	**40-64**	422,054 (42.97)	54 (48–59)	**2.57 ± 2.6**	**4.83 ± 2.42**	**258,615 (61.27)**	**512,975 (47.09)**
	**65-100**	229,949 (23.41)	72 (68–78)	**2.85 ± 2.58**	**5.13 ± 2.60**	**159,005 (69.15)**	**346,121 (51.90)**

N: number, SD: standard deviation, IQR: Interquartile Range.

*Polypharmacy: prescriptions with ≥ 5 medications.

Bold values indicate statistically significant differences (p < 0.001) for all comparisons between sexes and across age groups (t-test, ANOVA, or Chi-squared).

### Drug-drug interactions

**Fig 1:**
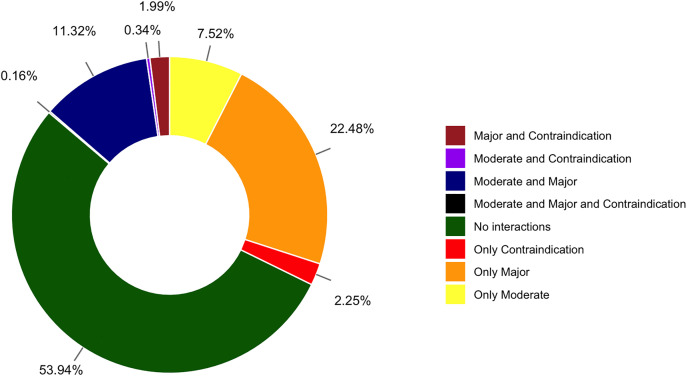
Prescription-Level Distribution of Drug–Drug Interaction Categories.

The five most frequently prescribed medications were acetaminophen (n = 422,819; 17.87% of all prescriptions), atorvastatin (303,720; 12.84%), azithromycin (269,454; 11.39%), acetylsalicylic acid (ASA, aspirin) (260,600; 11.02%), and the adult cold (acetaminophen + chlorpheniramine maleate + phenylephrine hydrochloride) (250,285; 10.58%). Outpatient prescriptions yielded 2,606,201 DDIs from 4,725 unique drug pairs, representing 28.59% of all possible pairs (16,529). Nearly half of prescriptions (46.06%, n = 1,089,718) contained at least one interaction, with an average of 2.39 ± 2.29 DDIs and 5.97 ± 2.58 drugs per prescription. At the patient level, 57.77% (n = 567,407) had at least one interacting prescription, averaging 1.92 ± 1.66 such prescriptions per patient. By severity, major interactions predominated (62.58%; n = 1,630,882), followed by moderate (32.82%; n = 855,443) and contraindicated (4.60%; n = 119,876). At the prescription level, 36.12% included at least one major DDI, 19.34% at least one moderate, and 4.73% at least one contraindicated combination. Detailed classifications are shown in Fig 1, with drug-specific analyses in Supplementary File 1 (S1 Table).

Fig 1 illustrates the proportion of all prescriptions assigned to eight mutually exclusive DDI categories. Over half of the prescriptions contained no interaction (dark green, 53.94%). Among those with DDIs, the largest segment consisted of prescriptions with only major interactions (orange, 22.48%), followed by those with both moderate and major interactions (dark blue, 11.32%) and only moderate interactions (yellow, 7.52%). Prescriptions containing only contraindicated interactions accounted for 2.25% (red). Less common patterns included major + contraindicated interactions (brown, 1.99%), moderate + contraindicated interactions (purple, 0.34%), and prescriptions featuring all three severities, moderate, major, and contraindicated (black, 0.16%).

### Most frequent drug-drug interactions

A limited number of drug pairs accounted for most DDIs. Among the 20 most prevalent interactions, 10 were moderate, eight major, and two contraindicated. The leading contraindicated pair was naproxen–ketorolac (1.77% of all interactions), with additional risks from other Non-steroidal anti-inflammatory drugs (NSAID)–ketorolac combinations (diclofenac, ibuprofen, celecoxib), highlighting duplicative NSAIDs use. Major interactions were dominated by acetylsalicylic acid, especially with metformin (2.36%), clopidogrel (1.46%), and hydrochlorothiazide (1.36%), reflecting frequent co-prescription of cardiovascular and antidiabetic agents. Moderate DDIs were likewise centered on acetylsalicylic acid, most often with metoprolol (2.25%), nitroglycerin (2.08%), and bisoprolol (1.17%). Table 2 summarizes the 10 most common DDIs, ranked by severity and frequency. The most frequent interactions involve agents with significant clinical safety profiles. For instance, the high prevalence of ASA + clopidogrel interactions carries a well-documented risk of major hemorrhage. Similarly, the ASA + metformin pair necessitates close monitoring for hypoglycemia, particularly in elderly populations. The top contraindicated pairs, predominantly NSAID duplications involving ketorolac, represent a high-risk prescribing pattern that significantly elevates the danger of acute kidney injury and gastrointestinal perforation. For a comprehensive drug-specific analysis Supplementary file 2 (S2 Table) provides detailed information on the top three interacting medications for each of the 100 most frequently prescribed drugs, stratified by interaction severity level.

**Table 2:** Top Drug-Drug Interactions by Severity and Frequency.

Interaction Severity	Target Drug	Interacting Agent	Repetitions	% of All Interactions
Moderate	Acetylsalicylic Acid	Metoprolol	58676	2.25
	Acetylsalicylic Acid	Nitroglycerin	54210	2.08
	Acetylsalicylic Acid	Bisoprolol	33023	1.27
	Atorvastatin	Clopidogrel	30572	1.17
	Levothyroxine	Metformin	28494	1.09
	Amlodipine	Metformin	28225	1.08
	Metformin	Metoprolol	27159	1.04
	Hydrochlorothiazide	Metformin	24350	0.93
	Empagliflozin	Gliclazide	23656	0.91
	Acetylsalicylic Acid	Carvedilol	19781	0.76
Major	Acetylsalicylic Acid	Metformin	61401	2.36
	Acetylsalicylic Acid	Clopidogrel	37956	1.46
	Acetylsalicylic Acid	Hydrochlorothiazide	35357	1.36
	Cetirizine	Diphenhydramine	26503	1.02
	Azithromycin	Famotidine	23353	0.90
	Famotidine	Ondansetron	22501	0.86
	Adult Cold	Codeine	21665	0.83
	Acetylsalicylic Acid	Furosemide	20762	0.80
	Naproxen	Dexamethasone	19000	0.73
	Losartan Potassium/ Hydrochlorothiazide	Acetylsalicylic Acid	17765	0.68
Contraindicated	Naproxen	Ketorolac	46201	1.77
	Diclofenac	Ketorolac	23011	0.88
	Ibuprofen	Ketorolac	15826	0.61
	Celecoxib	Ketorolac	13320	0.51
	Meloxicam	Ketorolac	7366	0.28
	Acetaminophen/Caffeine/ Ibuprofen	Ketorolac	6412	0.25
	Indomethacin	Ketorolac	2749	0.11
	Acetylsalicylic Acid	Ketorolac	2002	0.08
	Mefenamic	Ketorolac	1774	0.07
	Captopril	Valsartan	1403	0.05

**Fig 2:**
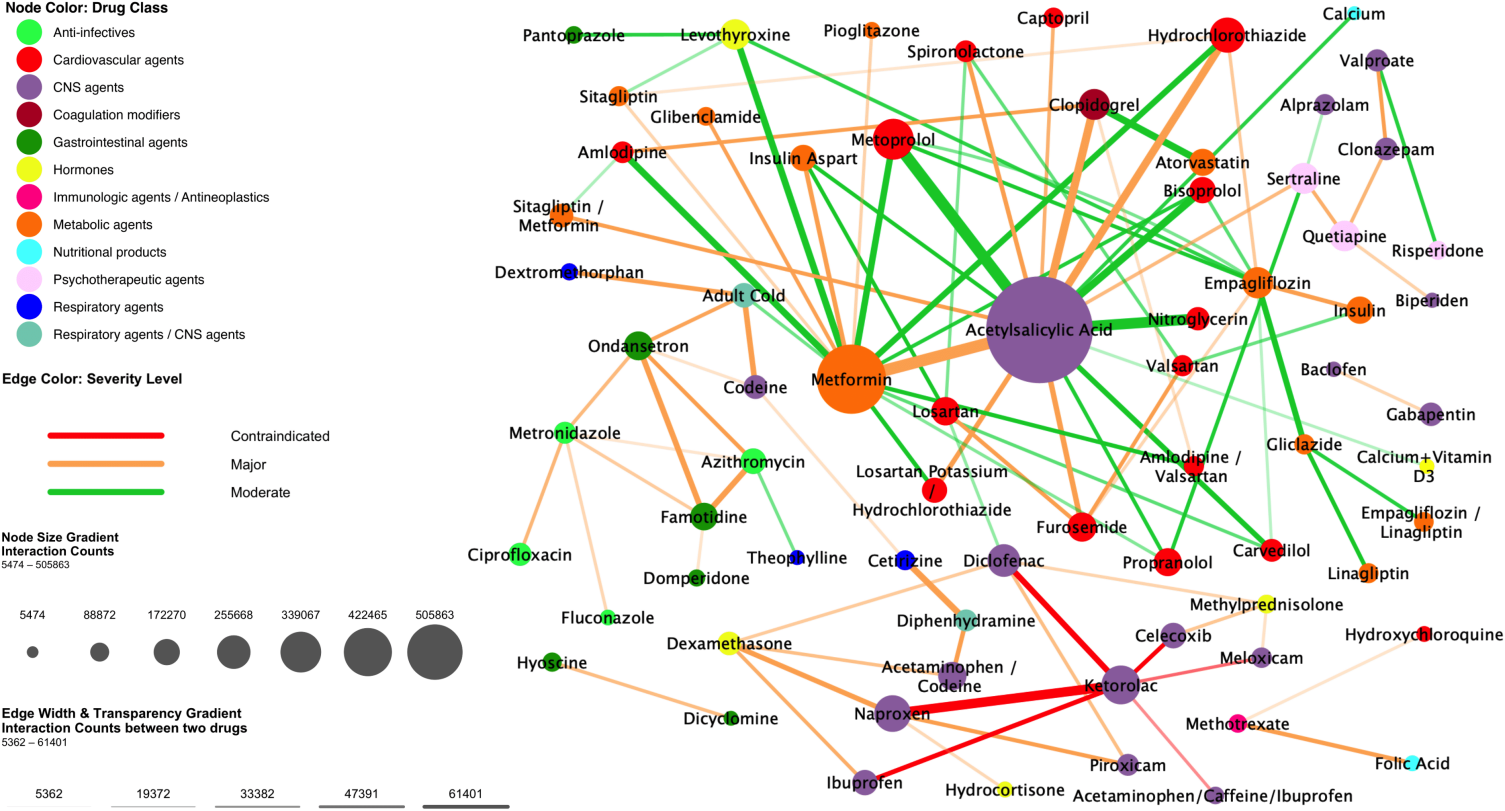
Network of High-Frequency Drug–Drug Interactions with Severity Coding.

Network analysis (Fig 2) depicts a densely connected network dominated by two interaction hubs, acetylsalicylic acid and metformin, whose node diameters denote the highest interaction burdens. The acetylsalicylic-acid–metformin pair shows the most significant edge weight, while similarly high-volume major interactions linked acetylsalicylic acid to clopidogrel and hydrochlorothiazide. Contraindicated interactions are confined to a compact NSAID cluster, with the thickest red edge between ketorolac and naproxen and additional red links involving diclofenac, ibuprofen, and celecoxib. Beyond these high-risk pairs, orange edges (major severity) connect cardiovascular, metabolic, and gastrointestinal agents, while the surrounding lattice of green edges (moderate severity) spans multiple therapeutic areas.

Fig 2 visualizes the top high-frequency drug–drug interactions detected in outpatient prescriptions. Node diameter is proportional to the total number of interactions attributed to each drug (larger nodes indicate higher interaction counts). Node color reflects the primary therapeutic class. Edge width is scaled to interaction frequency. Edge color denotes interaction severity—red = contraindicated, orange = major, green = moderate.

### Distribution of drug-drug interactions by sex and age

DDI prevalence was comparable between males (46.05%, n = 443,434) and females (46.07%, n = 646,284; p = 0.87). However, females had a higher rate of contraindicated prescriptions (4.94% vs. 4.43%), while males showed a greater mean number of interactions per prescription (1.15 vs. 1.07), driven by a higher prevalence of major (36.56% vs. 35.83%) and moderate (20.27% vs. 18.71%) interactions (all p < 0.001).

Age analysis revealed a progressive rise in DDI prevalence from children (0–18 years) (24.92%) to older adults (65–100 years) (52.48%), paralleled by an increase in mean interactions (0.38 ± 0.88 to 1.45 ± 2.31). Contraindicated interactions were highest in the 19–39-year group (7.27%), while major (39.62%) and moderate (30.27%) interactions peaked in the elderly (65–100 years) (all p < 0.001) (Table 3). Additionally, a comprehensive stratified analysis of the most frequent DDIs by sex and age groups is provided in Supplementary File 3 (S3 Table).

**Table 3:** Distribution and Severity of Drug–Drug Interactions Stratified by Sex and Age Group.

Variables		Total Prescriptions with DDIs (N, %)	Interactions per prescription (mean ± SD)	Prescriptions with Contraindications (N, %)	Prescriptions with Major DDIs (N, %)	Prescriptions with Moderate DDIs (N, %)
Sex	Male	443434 (46.05)	**1.15 ± 2.05**	**42656 (4.43)**	**352049 (36.56)**	**195169 (20.27)**
	Female	646284 (46.07)	**1.07 ± 1.90**	**69237 (4.94)**	**502664 (35.83)**	**262446 (18.71)**
Age groups (years)	0-18	**62882 (24.92)**	**0.38 ± 0.88**	**7107 (2.82)**	**55895 (22.15)**	**6322 (2.51)**
	19-39	**155365 (43.49)**	**0.87 ± 1.60**	**25973 (7.27)**	**129030 (36.12)**	**33476 (9.37)**
	40-64	**521482 (47.87)**	**1.13 ± 1.97**	**58087 (5.33)**	**405571 (37.23)**	**215959 (19.83)**
	65-100	**349989 (52.48)**	**1.45 ± 2.31**	**20726 (3.11)**	**264217 (39.62)**	**201858 (30.27)**

N: number, SD: standard deviation.

Bold values indicate statistically significant differences (p < 0.001) for all comparisons between sexes and across age groups (t-test, ANOVA, or Chi-squared).

### Drug-drug interactions by physician specialty

Among the ten highest-prescribing specialties, general practitioners issued the largest share of prescriptions, with more than half involving polypharmacy second only to cardiology. Despite this, DDI prevalence in general practice (43.09%) was lower than in several specialties. Cardiology showed the most intense DDI exposure: over two-thirds of prescriptions contained interactions (70.29%), with the highest mean drug count (5.60 ± 2.67) and average number of interactions per interacting prescription (3.34 ± 2.72). Psychiatry had a similarly high prevalence (81.99%) and the second-highest DDI average (3.24 ± 3.16), though with lower polypharmacy rate (37.63%). Pediatrics had the lowest DDI burden (20.14%) and fewer interactions per prescription (1.89 ± 1.76). Urology also recorded low polypharmacy (14.2%) and interaction prevalence (28.31%). In terms of severity, orthopedics (30.24%) and emergency medicine (21.74%) had the highest rates of contraindicated interactions, while neurology and psychiatry were dominated by major DDIs (≥ 90%). In contrast, moderate interactions were a particularly prominent feature in cardiology, where their prevalence surpassed that of major interactions (Table 4). A comprehensive list of the five most frequent interactions for each specialty and the three leading contraindicated, major, and moderate pairs can be found in supplementary file 4 (S4 Table).

**Table 4:** Prescription Volume, Polypharmacy, and Severity-Profiled Drug–Drug Interactions Across the Ten Highest-Volume Physician Specialties.

Specialty	Total Prescriptions (N, %)	Prescriptions with Polypharmacy (N, %)	Medication per prescriptions (mean ± SD)	Prescriptions with DDIs				
				**Total** **(N, %)**	**Interactions (mean ± SD)**	**Contraindication DDIs (N, %)**	**Major DDIs** **(N, %)**	**Moderate DDIs** **(N, %)**
**General practice**	995336 (42.07)	543468 (54.6)	5.21 **±** 2.46	428877 (43.09)	2.08 **±** 1.91	74470 (17.36)	328932 (76.7)	139558 (32.54)
**Internal medicine**	409664 (17.32)	196316 (47.92)	4.97 **±** 2.62	188118 (45.92)	2.54 **±** 2.47	4128 (2.19)	140105 (74.48)	102751 (54.62)
**Cardiology**	184366 (7.79)	113952 (61.81)	5.60 **±** 2.67	129590 (70.29)	3.34 **±** 2.72	1264 (0.98)	93636 (72.26)	111823 (86.29)
**Emergency medicine**	152665 (6.45)	75458 (49.43)	4.74 **±** 1.96	62797 (41.13)	1.85 **±** 1.62	13650 (21.74)	48889 (77.85)	13576 (21.62)
**Psychiatry**	100915 (4.27)	37978 (37.63)	4.32 **±** 2.11	82744 (81.99)	3.24 **±** 3.16	1160 (1.4)	76696 (92.69)	30618 (37)
**Orthopedics**	86003 (3.64)	24857 (28.90)	3.84 **±** 1.56	31060 (36.12)	1.61 **±** 1.20	9394 (30.24)	24443 (78.7)	2864 (9.22)
**Neurology**	83723 (3.54)	34572 (41.29)	4.56 **±** 2.33	53467 (63.86)	2.67 **±** 2.51	1714 (3.21)	48849 (91.36)	17653 (33.02)
**Obstetrics and Gynecology**	50681 (2.14)	11623 (22.93)	3.59 **±** 1.61	20055 (39.57)	1.6 **±** 1.30	156 (0.78)	17483 (87.18)	4685 (23.36)
**Pediatrics**	48956 (2.07)	14374 (29.36)	3.94 **±** 1.76	9862 (20.14)	1.89 **±** 1.76	260 (2.64)	8163 (82.77)	3176 (32.2)
**Urology**	38257 (1.62)	5429 (14.2)	3.19 **±** 1.49	10831 (28.31)	1.60 **±** 1.28	569 (5.25)	7931 (73.23)	4633 (42.78)

DDI: drug-drug interaction, N: number, SD: standard deviation.

### Independent Predictors of Drug–Drug Interaction Count

**Fig 3:**
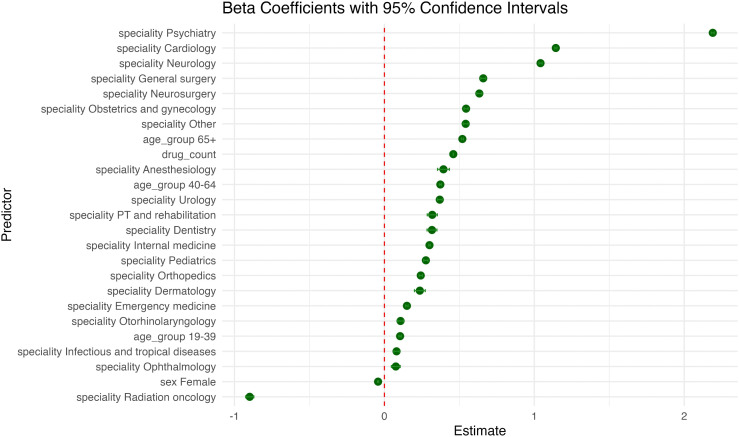
Multivariable linear regression for predictors of the number of DDIs in prescriptions.

After excluding specialties with fewer than 5,000 prescriptions (constituting roughly 0.5% of all prescriptions) to improve representativeness and stability of estimates, multivariable linear regression identified several independent predictors of prescription-level DDI counts. The number of drugs per prescription remained the dominant predictor. Each additional medication increased the expected interaction count by β = 0.460 (95% CI 0.459–0.461; p < 0.001). Physician specialty showed the widest effect range. Compared with general practitioners, prescriptions written by psychiatrists had the largest incremental DDI load (β = 2.191, 95% CI 2.181 to 2.200; p < 0.001), followed by cardiology (β = 1.143, 95% CI 1.136 to 1.151, p < 0.001) and neurology (β = 1.042, 95% CI 1.031 to 1.052, p < 0.001). Conversely, radiation oncology was associated with a significant decrease in DDI load (β = −0.899, 95% CI −0.926 to −0.871; p < 0.001). Age demonstrated a graded positive association. Relative to children (0–18 years), prescriptions for adults aged 19–39, 40–64, and 65–100 years increased DDI numbers by β = 0.105 (95% CI 0.097–0.113), β = 0.373 (95% CI 0.366–0.380), and β = 0.520 (95% CI 0.513–0.528), respectively (all p < 0.001). Sex had a modest inverse effect, with female prescriptions showing β = –0.042 (95% CI –0.046 to –0.038; p < 0.001) compared with males. The model accounted for 40.2% of the variance in interaction counts (adjusted R² = 0.402). Fig 3 illustrates the relative magnitude and direction of each predictor’s effect on the number of DDIs.

Plot of standardized β coefficients (dots) with 95% confidence intervals (horizontal bars) for predictors of prescription-level drug–drug interaction counts in the multivariable linear regression model. Estimates are shown relative to their reference categories: male sex, age 0–18 years, and general practice. The vertical dashed line at β = 0 denotes no association; coefficients to the right indicate an increase and those to the left a decrease in the expected number of interactions.

**Fig 4:**
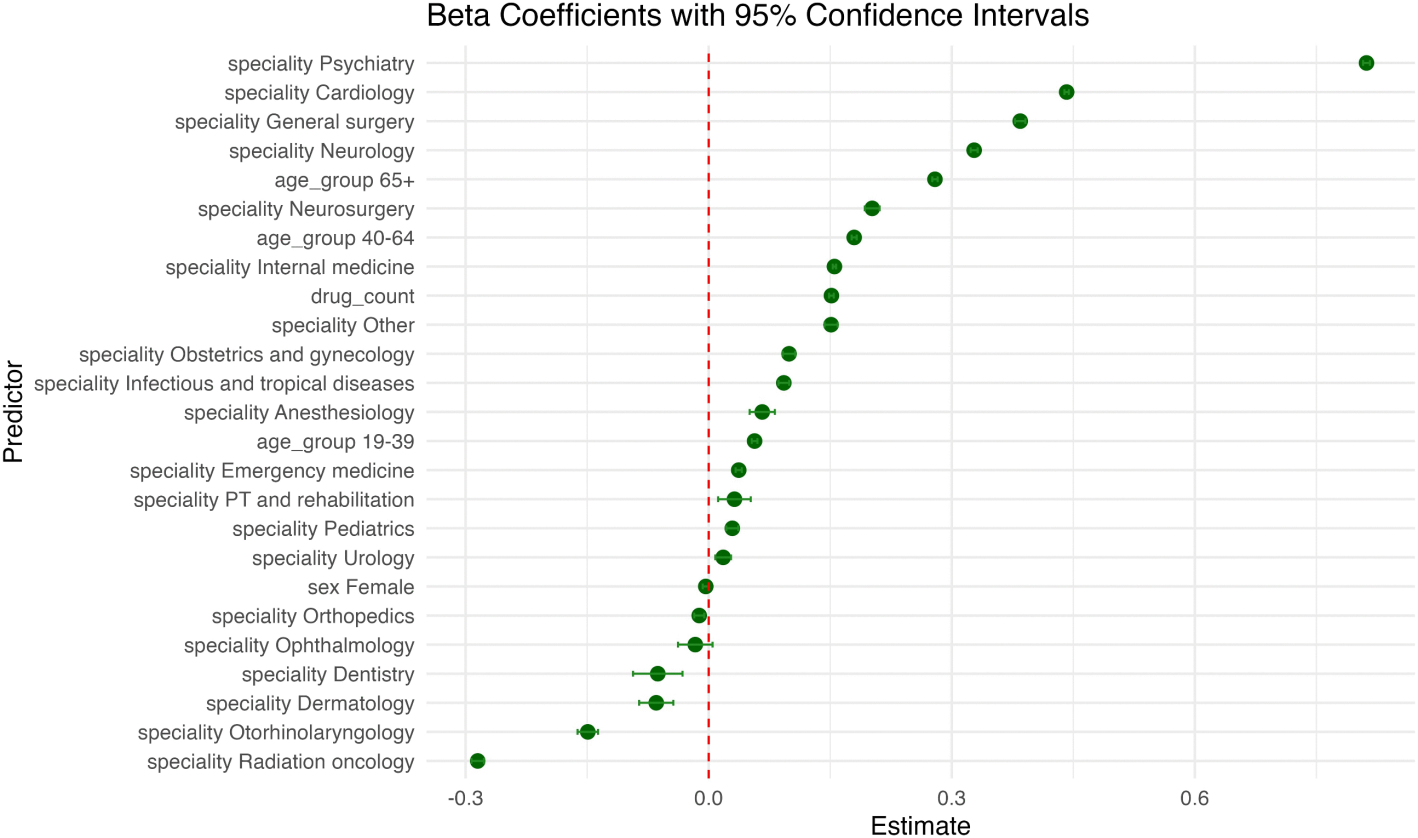
Interaction-Adjusted Effects of Predictors on Drug–Drug Interaction Counts.

In the interaction-adjusted linear model, the incremental effect of drug count on DDI burden differed markedly across age groups and physician specialties. The baseline slope for drug count was β = 0.152 (95% CI 0.149–0.154; p < 0.001); however, this slope increased progressively with patient age, rising by an additional 0.057, 0.180, and 0.279 units in the 19–39, 40–64, and ≥ 65-year groups, respectively (all p < 0.001). A comparable pattern emerged across specialties: relative to general practice, the drug-count slope was steepest in psychiatry (β = 0.812, 95% CI 0.808–0.816) and remained elevated in cardiology (β = 0.442, 95% CI 0.440–0.445), general surgery (β = 0.385, 95% CI 0.378–0.391), and neurology (β = 0.328, 95% CI 0.323–0.332) (all p < 0.001). Sex did not significantly modify the drug-count effect (β = –0.004, p = 0.063). Incorporation of these interaction terms improved model fit, yielding an adjusted R² of 0.477. Interaction effects between drug count and key predictors are illustrated in Fig 4.

This figure presents the estimated coefficients and 95% confidence intervals from a multivariable linear regression model including interaction terms between drug count and key predictors. The vertical dashed line at zero indicates no effect. Positive coefficients reflect a greater increase in DDIs per additional medication, conditional on age group or physician specialty. Notably, interaction slopes were steepest for psychiatry, cardiology, and older age groups, indicating a stronger marginal effect of drug count in these subgroups.

### Risk factors for contraindicated medication combinations

**Fig 5:**
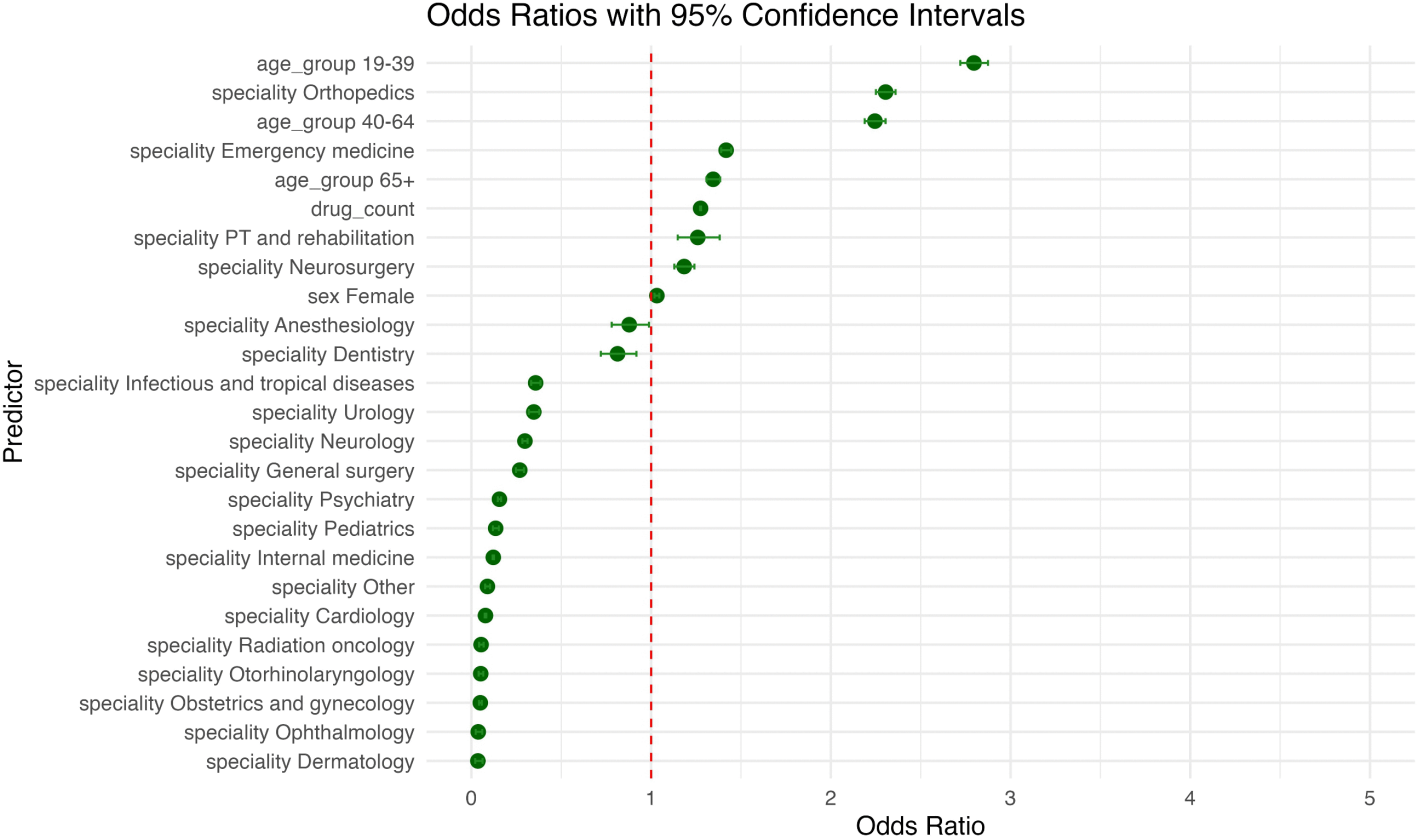
Estimated Effects of Predictors on the Likelihood of Contraindicated Drug–Drug Interactions.

In the multivariable logistic regression model predicting the presence of contraindicated DDIs, physician specialty emerged as the strongest predictor. Compared with general practitioners, prescriptions issued by orthopedic specialists had the highest odds of containing contraindications (OR = 2.305, 95% CI 2.251–2.360; p < 0.001), followed by emergency medicine (OR = 1.417, 95% CI 1.390–1.446; p < 0.001) and physical therapy and rehabilitation (OR = 1.260, 95% CI 1.148–1.381; p < 0.001). In contrast, specialties such as dermatology (OR = 0.036, 95% CI 0.021–0.062; p < 0.001), ophthalmology (OR = 0.038, 95% CI 0.024–0.060; p < 0.001), and obstetrics and gynecology (OR = 0.050, 95% CI 0.042–0.058; p < 0.001) were associated with significantly reduced odds of contraindicated prescriptions. Drug count was another strong and independent predictor. Each additional medication increased the odds of a contraindicated interaction by 27.5% (OR = 1.275, 95% CI 1.272–1.278; p < 0.001). Age also showed a graded association with contraindications. Compared to individuals aged 0–18 years, those aged 19–39 years had the highest odds (OR = 2.796, 95% CI 2.720–2.874; p < 0.001), followed by 40–64 years (OR = 2.245, 95% CI 2.188–2.304; p < 0.001) and 65–100 years (OR = 1.345, 95% CI 1.307–1.384; p < 0.001). Female sex was associated with a modest but statistically significant increase in the likelihood of contraindicated interactions compared to males (OR = 1.032, 95% CI 1.019–1.046; p < 0.001). The model demonstrated good overall fit (AIC = 766625), with specialty and drug count identified as the most influential predictors of contraindication presence. Fig 5 shows the direction and magnitude of each predictor’s influence on contraindicated DDIs.

This figure presents the odds ratio and 95% confidence intervals from the multivariable logistic regression model assessing the association between patient and physician characteristics and the presence of contraindicated DDIs. Estimates are shown relative to the reference categories: male sex, age group 0–18 years, and general practice. Predictors with positive coefficients increased the likelihood of contraindications, while those with negative coefficients were associated with reduced odds.

## Discussion

We identified a high burden of potential drug–drug interactions (DDIs) in routine outpatient care in Iran, with nearly half of all prescriptions (46.1%) containing at least one DDI, with a mean of 2.39 interactions. In total, we identified 4,725 distinct drug–drug pairs involved in DDIs, though a small number of combinations (notably involving cardiovascular, metabolic, and analgesic agents) accounted for most cases. For example, acetylsalicylic acid (ASA), also known as aspirin, appeared in >17% of all major DDIs, and NSAIDs like ketorolac dominated the contraindicated list. Psychiatry (81.99%) and cardiology (70.29%) had the highest rates of DDI-positive prescriptions. Although orthopedic and emergency medicine had lower overall DDI rates, they accounted for the highest proportion of contraindicated interactions. Multivariable analysis confirmed polypharmacy, advanced age, and (to a lesser extent) male sex as independent DDI predictors. Each additional drug raised the expected number of the DDIs by roughly 0.4, and this effect was strongest in the elderly and prescriptions of psychiatrists.

The overall DDI prevalence in this nationwide outpatient cohort (46.06% of prescriptions) is remarkably higher relative to earlier Iranian reports. For example, Nabovati et al. (18) conducted a systematic review of 21 studies in Iran, finding a median DDI rate of only 8.5% in outpatient prescriptions (and 19.2% in inpatients). However, the study quality was generally poor (many small, single-center studies with narrow drug lists). Other Iranian studies report a wide range of outpatient DDI prevalence, which varies depending on the population, clinical setting, methodology, and analysis strategy. For instance, Dirin et al. [21] (2014) analyzed 2,796 prescriptions from a teaching hospital in eastern Iran, finding 41.6% of prescriptions contained ≥1 potential DDI, whereas Ahmadizar et al. [22] used a very large national sample (about 44 million prescriptions in 2007–2008) and strict criteria (Drug Interaction Facts); they reported only 0.77% of scripts had any DDI. Similarly, Mousavi et al. [23] analyzed 1260 prescriptions from community and outpatient hospital pharmacies in Tehran, reporting a DDI prevalence of 26.9%, of which 7.3% were major or contraindicated. A large provincial study in Khorasan Razavi, evaluating 8.17 million prescriptions, identified 6,096 clinically relevant potential DDIs (0.07%), especially in cardiology and internal medicine [24]. In pediatrics, Asadi et al. [25] found that 26.7% of 1,011 outpatient prescriptions contained at least one clinically significant potential DDI, predominantly moderate in severity and involving risk category C [26], with patients having underlying conditions facing an 80% higher interaction risk. A post-PCI (percutaneous coronary intervention) cardiology cohort in Shiraz had 82.8% DDI prevalence, largely due to dual antiplatelet therapy plus ACE (Angiotensin-converting enzyme) inhibitors [27]. These discrepancies are likely due to methodological differences. Our study analyzed over 2.3 million insurance records, focusing on the 100 most frequently used medications, and applied Micromedex severity grading. This comprehensive approach naturally increased the likelihood of detecting DDIs. As Nabovati et al. highlighted, most prior Iranian studies suffered from variable definitions and low methodological quality [28].

High outpatient DDI rates are not unique to Iran. In Uganda, Lule et al. screened 295 prescriptions at private pharmacies and found 37.6% had ≥ 1 potential DDI [3]. In one Jordanian polypharmacy clinic, 96% of patients had ≥ 1 potential DDI [29], while 57.6% of prescriptions carried potential DDIs in an Iraqi outpatient series [30]. A cross-sectional study in Pakistan also showed that 22.3% of outpatients had potential DDIs in a tertiary care hospital [4]. Another Brazilian primary-care study of 827 patients reported 63.0% had ≥ 1 potential DDI (12.1% major DDIs) [31]. These LMIC examples are comparable to our 46% and highlight how age, comorbidity, and drug use drive high DDI prevalence. Even high-income countries experience substantial DDI prevalence, though rates vary by methodology. For example, a Slovenian nationwide claims study (over 1.1 million outpatients) found 24.1% of the population exposed to potential DDIs (9.3% major), with higher rates among women and the elderly [32]. Similarly, 30.3% potential DDIs (8.5% severe) were reported in 16,120 Chinese hospital outpatient orders [33]. In summary, 30–60% DDI prevalence is common worldwide when polypharmacy is prevalent.

An important observation in our study is the distribution of DDI severity. Major interactions comprised 62.6% of all DDIs, while moderate interactions accounted for 32.8%. This differs from some previous reports, where moderate interactions were more common. In a Pakistani outpatient study, moderate DDIs represented 62% compared to 32% major [4], and another study in Iraq found that about half were moderate and around 40% major [30]. This likely reflects the drugs most often prescribed in Iran.

The most frequent major DDIs included ASA+metformin, ASA+clopidogrel, ASA+hydrochlorothiazide, ASA+furosemide, and other cardiometabolic pairs (Table 2). ASA appeared in several top major interactions, expected since it is widely used and combines with many drugs to raise bleeding or other risks [34–37]. However, while combinations such as ASA and clopidogrel are clinically indicated as standard dual antiplatelet therapy, they were classified as ‘Major’ interactions in this study according to Micromedex criteria and due to the significantly increased risk of hemorrhage, which requires vigilant clinical monitoring. This does not necessarily mean these combinations should be avoided in clinical settings. Similar findings have been reported in other studies; for instance, a Brazilian hospital study showed cardiovascular drugs strongly linked to DDIs [38]. Ulfa et al. in Indonesia also documented a 42.1% DDI prevalence among chronic disease patients, with cardiovascular and analgesic drugs being the most frequently involved combinations [39].

The contraindicated DDIs in our data mostly involved duplicate NSAIDs. Clinical guidelines recommend avoiding the use of more than one NSAID at the same time [40,41]. When compared with other studies, our results are consistent in highlighting cardiovascular and pain-relief drugs as the most frequent sources of DDIs [30,38,39,42].

Our results align with findings from other studies, where multimorbidity in older adults leads to complex regimens and increased risk of DDIs [43–45]. Age-related declines in renal and hepatic function, along with more chronic conditions and polypharmacy, increase both exposure to and vulnerability to DDIs (supplementary table 3) [45,46].

Prescription-level DDI prevalence was nearly identical between sexes. Males had a slightly higher mean number of interactions per affected prescription. This small effect aligns with other large cohort studies [47,48] and was confirmed in our own analysis, where the effect of gender on the number of interactions became non-significant after adjusting for the number of medications per prescription and other covariates.

Regarding severity, contraindications were somewhat more common in female patients, whereas major DDIs were slightly more frequent in males. These findings may be partly explained by prescribing patterns observed in our cohort (Supplementary Table 3). The higher prevalence of major DDIs in males could be linked to a greater use of medications for chronic metabolic and cardiac conditions, while the higher rate of contraindications in females may be associated with more frequent prescribing of analgesic drugs. These patterns are consistent with prior pharmacovigilance data suggesting sex-related differences in drug metabolism and prescribing [49].

DDI prevalence varied widely across specialties. Despite having one of the lowest polypharmacy rates, psychiatry had the highest DDI prevalence. This likely reflects the nature of psychiatric prescribing, where patients often take complex psychotropic regimens alongside other medications [50]. Cardiology prescriptions averaged 5.60 medications, and 70.3% had DDIs, consistent with complex cardiovascular regimens (antiplatelets, anticoagulants, antihypertensives, statins, etc.). Previous literature emphasizes that psychiatric polypharmacy is a major driver of interactions [50,51], and cardiology patients often require multiple interacting agents [22]. The significant reduction in the number of drug–drug interactions (DDIs) in prescriptions from radiation oncology specialists can be attributed to the specific prescribing patterns of this field. In our analysis, the most common outpatient drugs prescribed by these specialists were antiemetics, which are generally associated with a low risk of drug interactions (supplementary table 4). Interestingly, orthopedics and emergency medicine were in the middle for overall DDI, but they had the highest proportions of contraindicated interactions. This suggests that there was more frequent co-prescribing of prohibited NSAID combinations. Indeed, ketorolac and similar NSAIDs are commonly used in emergency departments and orthopedic wards for acute pain [52–54], which appears to have led to more cases of overlapping NSAID orders. The finding that Dermatology and Ophthalmology had the lowest odds of contraindicated prescriptions can be explained by their extensive use of topical agents. These medications typically have minimal systemic absorption, which reduces their potential for causing significant drug-drug interactions.

In our analysis, polypharmacy was the strongest predictor; each additional drug added approximately 0.46–0.52 to the expected DDI count. This is consistent with prior studies showing approximately 40% higher DDI risk per extra drug [55,56]. Polypharmacy is strongly linked to ADRs, hospitalizations, and reduced medication adherence [57,58]. In a UK study, patients receiving ≥10 drugs were five times more likely to experience a DDI than those prescribed 2–4 drugs [59]. In Korea, polypharmacy among elderly stroke survivors was associated with adverse outcomes such as falls and mortality [60]. Similar studies from Saudi Arabia and Indonesia have found polypharmacy rates ranging from 42–70% in outpatient settings, and the most important risk factors associated with the DDIs [61–63]. The Swedish Prescribed Drug Register study also showed that the probability of any DDI rose steeply with each extra medication [56], and DeRemer [55], reported a 40–50% increase in DDI risk per added drug in cancer patients. Guthrie *et al.* [59] similarly observed that patients on ≥10 drugs were about five times more likely to have DDIs than those on 2–4 drugs.

We further explored how polypharmacy’s impact on DDIs varied by age and specialty using an “interaction-adjusted” model. This showed that the marginal effect of each extra drug is not constant but grows with patient age and certain specialties. Practically, prescribing one more medication to a 70-year-old adds far more DDI risk than to a 10-year-old. Elderly patients already have complex regimens and limited reserves, so incremental danger is magnified, aligning with the concept that frail or multimorbid patients suffer disproportionately from each additional therapy [64]. Specialty also influenced how strongly additional drugs increased DDI risk. Psychiatry showed the steepest rise, with each added drug rapidly increasing interactions, reflecting psychotropic polypharmacy complexity, where even a few agents can interact in multiple ways. Cardiology, neurology, and general surgery also demonstrated elevated slopes, emphasizing overlapping mechanisms and dense interaction networks.

The results regarding the prevalence of contraindications in the young population may reflect prescribing behavior; clinicians may be more cautious in avoiding strict contraindications in the elderly, while younger adults more often receive aggressive polypharmacy (multiple painkillers or over-the-counter drugs (OTCs)), leading to contraindications [65].

Our findings call for a multifaceted approach, beginning with nationwide adoption of electronic prescribing (eRx) systems integrated with clinical decision support systems (CDSS), shown to reduce DDIs via real-time alerts for high-risk combinations such as ketorolac with other NSAIDs or ASA with clopidogrel [66–68]. Continuing medical education (CME) should address DDI mechanisms, common high-risk combinations, and safer alternatives, particularly in high-risk specialties. Greater integration of clinical pharmacists into outpatient care through medication therapy management can intercept interactions before dispensing [21]. Patient education can also improve awareness and reduce unsupervised use of OTC drugs, particularly NSAIDs [69]. Health authorities should enforce stricter reimbursement policies, such as limiting coverage of duplicate NSAIDs or requiring justification for high-risk combinations, and establish a national DDI registry to monitor trends.

### Strengths and limitations

This study offers one of the most comprehensive evaluations of outpatient DDIs in Iran. We used a large-scale dataset (over 2.3 million prescriptions). DDIs were classified using the Micromedex system, ensuring consistency and comparability. We applied robust regression models adjusting for demographics and specialty, isolating independent risk factors. We also conducted a detailed analysis stratified by age, sex, and specialty, providing practical insight into subpopulation risks. Despite its strengths, the study has limitations. It reports potential DDIs without measuring actual clinical harm, such as hospitalizations or adverse outcomes. Our dataset includes only IHIO-prescribed medications. Common OTC drugs (especially NSAIDs) and herbal remedies were not captured, so the DDI burden may be higher. Furthermore, our analysis was restricted to the 100 most frequently prescribed medications. While this approach focuses on the drugs responsible for the largest DDI burden at a national level, it may have missed rare but clinically relevant interactions involving less common agents. Additionally, our prevalence estimates are limited to moderate, major, and contraindicated interactions to prioritize clinical relevance over absolute frequency. Although this approach likely yields a lower DDI rate than studies including minor interactions it offers a more conservative and policy-focused assessment of the risks requiring immediate intervention. Adherence and exact medication duration were not accounted for. By design, we only analyzed multi-drug prescriptions, so potential interactions involving a physician’s script plus a patient’s OTC drug (e.g., ibuprofen) are missed. We looked at prescriptions for one year. We cannot assess longitudinal outcomes (e.g., if a given DDI leads to hospitalization) or changes over time. Moreover, the study was indifferent to the certainty of evidence regarding Micromedex categories. While Micromedex is a highly reliable, evidence-based resource, we acknowledge that DDI classifications can vary across different databases. Exclusive reliance on one tool may introduce source-specific bias. Also, regression models accounted for the primary demographic and prescribing confounders available in the insurance database, such as age, sex, and polypharmacy. However, the lack of data on clinical comorbidities and socioeconomic status remains a limitation that may influence the identified DDI risks. While this study establishes a baseline for DDI prevalence, future research should move toward more granular, stratified analyses. Specifically, investigating interaction patterns within high-risk drug classes and narrower age brackets could identify specific clinical vulnerabilities.

## Conclusion

This large-scale analysis highlights a high burden of potential DDIs in Iranian outpatient prescriptions, particularly among elderly patients, men, and those receiving multiple medications. The dominance of major interactions and the presence of over 100,000 contraindicated prescriptions underscore the urgent need for comprehensive prescribing reform. Polypharmacy and age are the principal drivers, and a few common drug classes (NSAIDs, antiplatelets, antidiabetics) dominate the risk. Health systems should prioritize e-prescribing, clinician education, pharmacist integration, and patient engagement to mitigate DDI risks and improve medication safety.

## Supporting information

S1 Table Comprehensive Analysis of Drug Interaction Proportions Among the 100 Most Frequently Prescribed Medications in the IHIO Database. (DOCX)

S2 Table Top Three Interacting Drugs for Each of the 100 Most Frequently Prescribed Medications, Stratified by Interaction Severity. This table presents the three most frequent drug interaction partners for each of the 100 most commonly prescribed medications in the IHIO database, categorized by severity level (contraindicated, major, and moderate). Numbers represent the absolute frequency of co-prescription, while percentages (in parentheses) indicate the proportion relative to all prescriptions containing the target drug listed in the first column. Empty cells indicate no interactions were identified in that severity category.
(DOCX)

S3 Table Distribution of Top Drug-Drug Interactions by Severity Level Across Sex, Age Groups. (DOCX)

S4 Table Specialty-Specific Drug–Drug Interaction Profiles: Top 5 Interactions Overall and Top 3 Interactions Within Each Severity Category (Major, Moderate, Contraindicated). (DOCX)

## Abbreviations

DDIs: Drug–drug interactions
LMICs: Low and middle-income countries
IHIO: Iran Health Insurance Organization
IQR: Interquartile range
OR: Odds ratios
CI: Confidence intervals
NSAIDs: Non-steroidal anti-inflammatory drugs
PCI: Percutaneous coronary intervention
ACE: Angiotensin-converting enzyme
ASA: Acetylsalicylic acid
CDSS: Clinical decision support systems
CME: Continuing medical education
OTCs: Over-the-counter drugs
GP: General practitioner
