# Supplementary material for: Prevalence, predictors, and clinical relevance of drug–drug interactions in outpatient prescribing: A national cross-sectional study
Source: PLoS One. 2026 Apr 8;21(4):e0345076. doi: 10.1371/journal.pone.0345076 (PMC13061183; doi:10.1371/journal.pone.0345076)
Supplement: S1 Table — (DOCX) [file pone.0345076.s001.DOCX]

**S1 Table1.** Comprehensive Analysis of Drug Interaction Proportions Among the 100 Most Frequently Prescribed Medications in the IHIO Database

.

| **Name** | **Micromedex interaction pairs** | | **Target drug (% of total drugs)** | | **Prescriptions with target drug (% of total prescriptions)** | | **Average drug count per prescription** | | **Number of all interactions found** | | **Interaction pairs found (% of total possible interaction pairs)** | | **Prescriptions with at least one interaction (% of all prescriptions containing the target drug)** | | **Average interaction number per prescriptions containing interactions of that target drug)** | |
| --- | --- | --- | --- | --- | --- | --- | --- | --- | --- | --- | --- | --- | --- | --- | --- | --- |
| **Acetaminophen** | | 18 | | 467962 (4.07%) | | 422819 (17.87%) | | 5.92 | | 1010 | | 6 (33.33%) | | 985 (0.23%) | | 1.025 |
| **Atorvastatin** | | 126 | | 304540 (2.65%) | | 303720 (12.84%) | | 6.16 | | 75658 | | 41 (32.54%) | | 68371 (22.51%) | | 1.107 |
| **Azithromycin** | | 232 | | 270293 (2.35%) | | 269454 (11.39%) | | 5.73 | | 67880 | | 91 (39.22%) | | 57574 (21.37%) | | 1.179 |
| **Acetylsalicylic Acid** | | 364 | | 260752 (2.27%) | | 260600 (11.02%) | | 6.99 | | 505863 | | 115 (31.59%) | | 224028 (85.97%) | | 2.258 |
| **Adult Cold** | | 126 | | 251035 (2.18%) | | 250285 (10.58%) | | 5.96 | | 63007 | | 47 (37.30%) | | 57316 (22.90%) | | 1.099 |
| **Vitamin B1** | | 5 | | 247890 (2.16%) | | 237183 (10.03%) | | 5.95 | | 132 | | 2 (40.00%) | | 132 (0.06%) | | 1 |
| **Pantoprazole** | | 73 | | 239124 (2.08%) | | 232775 (9.84%) | | 6.43 | | 24602 | | 33 (45.21%) | | 22662 (9.74%) | | 1.086 |
| **Metformin** | | 315 | | 227441 (1.98%) | | 220637 (9.33%) | | 5.83 | | 303878 | | 99 (31.43%) | | 156081 (70.74%) | | 1.947 |
| **Losartan** | | 114 | | 212109 (1.84%) | | 210405 (8.89%) | | 6.11 | | 82597 | | 38 (33.33%) | | 64146 (30.49%) | | 1.288 |
| **Famotidine** | | 161 | | 163842 (1.42%) | | 163013 (6.89%) | | 6.22 | | 78427 | | 58 (36.02%) | | 68052 (41.75%) | | 1.152 |
| **Ondansetron** | | 313 | | 159252 (1.38%) | | 123274 (5.21%) | | 6.25 | | 85773 | | 104 (33.23%) | | 62396 (50.62%) | | 1.375 |
| **Naproxen** | | 258 | | 153252 (1.33%) | | 153112 (6.47%) | | 5.87 | | 127680 | | 80 (31.01%) | | 90005 (58.78%) | | 1.419 |
| **Cefixime** | | 18 | | 138081 (1.20%) | | 137966 (5.83%) | | 5.34 | | 295 | | 7 (38.89%) | | 270 (0.20%) | | 1.093 |
| **Gabapentin** | | 171 | | 125190 (1.09%) | | 124360 (5.26%) | | 5.15 | | 56698 | | 54 (31.58%) | | 41793 (33.61%) | | 1.357 |
| **Montelukast** | | 14 | | 123179 (1.07%) | | 123143 (5.21%) | | 6.16 | | 352 | | 7 (50.00%) | | 343 (0.28%) | | 1.026 |
| **Valsartan** | | 104 | | 118838 (1.03%) | | 117393 (4.96%) | | 6.13 | | 43786 | | 25 (24.04%) | | 34668 (29.53%) | | 1.263 |
| **Amlodipine** | | 81 | | 118282 (1.03%) | | 117669 (4.97%) | | 6.23 | | 46590 | | 29 (35.80%) | | 42368 (36.01%) | | 1.1 |
| **Diclofenac** | | 264 | | 117846 (1.02%) | | 112679 (4.76%) | | 5.31 | | 106057 | | 82 (31.06%) | | 68634 (60.91%) | | 1.545 |
| **Rosuvastatin** | | 94 | | 106800 (0.93%) | | 106544 (4.50%) | | 6.11 | | 6321 | | 15 (15.96%) | | 6190 (5.81%) | | 1.021 |
| **Chlordiazepoxide** | | 106 | | 106503 (0.93%) | | 105547 (4.46%) | | 5.26 | | 24017 | | 36 (33.96%) | | 20421 (19.35%) | | 1.176 |
| **Levothyroxine** | | 125 | | 105654 (0.92%) | | 105141 (4.44%) | | 5.62 | | 93506 | | 57 (45.60%) | | 53766 (51.14%) | | 1.739 |
| **Hydrochlorothiazide** | | 190 | | 102376 (0.89%) | | 101452 (4.29%) | | 5.96 | | 119320 | | 63 (33.16%) | | 68393 (67.41%) | | 1.745 |
| **Ibuprofen** | | 257 | | 99729 (0.87%) | | 98946 (4.18%) | | 5.39 | | 66860 | | 72 (28.02%) | | 45907 (46.40%) | | 1.456 |
| **Metoprolol Tartrate** | | 185 | | 94105 (0.82%) | | 94103 (3.98%) | | 6.44 | | 119418 | | 61 (32.97%) | | 68897 (73.21%) | | 1.733 |
| **Nitroglycerin** | | 13 | | 90096 (0.78%) | | 88277 (3.73%) | | 7.26 | | 55332 | | 5 (38.46%) | | 54709 (61.97%) | | 1.011 |
| **Folic Acid** | | 16 | | 89934 (0.78%) | | 89322 (3.78%) | | 6.35 | | 15216 | | 5 (31.25%) | | 15104 (16.91%) | | 1.007 |
| **Ciprofloxacin** | | 381 | | 88333 (0.77%) | | 86323 (3.65%) | | 4.39 | | 54070 | | 135 (35.43%) | | 35579 (41.22%) | | 1.52 |
| **Metronidazole** | | 231 | | 87913 (0.76%) | | 86896 (3.67%) | | 4.67 | | 48025 | | 86 (37.23%) | | 39279 (45.20%) | | 1.223 |
| **Empagliflozin** | | 79 | | 84787 (0.74%) | | 84129 (3.56%) | | 6.36 | | 98139 | | 29 (36.71%) | | 62841 (74.70%) | | 1.562 |
| **Cetirizine** | | 164 | | 83894 (0.73%) | | 83836 (3.54%) | | 5.86 | | 37200 | | 42 (25.61%) | | 33815 (40.33%) | | 1.1 |
| **Co-amoxiclav** | | 45 | | 83242 (0.72%) | | 83119 (3.51%) | | 4.96 | | 751 | | 13 (28.89%) | | 735 (0.88%) | | 1.022 |
| **Amoxicillin** | | 45 | | 80421 (0.70%) | | 80346 (3.40%) | | 4.78 | | 590 | | 14 (31.11%) | | 577 (0.72%) | | 1.023 |
| **Propranolol** | | 241 | | 78462 (0.68%) | | 78305 (3.31%) | | 5.39 | | 80553 | | 93 (38.59%) | | 52472 (67.01%) | | 1.535 |
| **Prednisolone** | | 191 | | 78427 (0.68%) | | 77608 (3.28%) | | 6.06 | | 31212 | | 41 (21.47%) | | 24573 (31.66%) | | 1.27 |
| **Hyoscine** | | 168 | | 73964 (0.64%) | | 60684 (2.57%) | | 6.06 | | 33345 | | 65 (38.69%) | | 23443 (38.63%) | | 1.422 |
| **Celecoxib** | | 299 | | 72820 (0.63%) | | 72781 (3.08%) | | 4.76 | | 68115 | | 97 (32.44%) | | 42315 (58.14%) | | 1.61 |
| **Omeprazole** | | 110 | | 70988 (0.62%) | | 70983 (3.00%) | | 5.69 | | 18372 | | 38 (34.55%) | | 15460 (21.78%) | | 1.188 |
| **Sertraline** | | 435 | | 70747 (0.62%) | | 70320 (2.97%) | | 5.18 | | 96133 | | 132 (30.34%) | | 53667 (76.32%) | | 1.791 |
| **Gliclazide** | | 16 | | 67479 (0.59%) | | 67067 (2.83%) | | 5.98 | | 39272 | | 7 (43.75%) | | 27272 (40.66%) | | 1.44 |
| **Bisoprolol Fumarate** | | 153 | | 66548 (0.58%) | | 66446 (2.81%) | | 6.48 | | 71043 | | 45 (29.41%) | | 44806 (67.43%) | | 1.586 |
| **Acetaminophen / Codeine** | | 460 | | 64690 (0.56%) | | 64644 (2.73%) | | 6.01 | | 82711 | | 136 (29.57%) | | 48440 (74.93%) | | 1.707 |
| **Furosemide** | | 208 | | 60861 (0.53%) | | 54990 (2.32%) | | 7.52 | | 86620 | | 74 (35.58%) | | 44125 (80.24%) | | 1.963 |
| **Clopidogrel** | | 307 | | 59761 (0.52%) | | 59761 (2.53%) | | 7.25 | | 96649 | | 77 (25.08%) | | 52196 (87.34%) | | 1.852 |
| **Acetylcysteine** | | 2 | | 59550 (0.52%) | | 59506 (2.52%) | | 6.55 | | 1124 | | 2 (100.00%) | | 1107 (1.86%) | | 1.015 |
| **Valproate** | | 75 | | 56848 (0.49%) | | 56667 (2.40%) | | 5.29 | | 50265 | | 41 (54.67%) | | 35177 (62.08%) | | 1.429 |
| **Amlodipine / Valsartan** | | 188 | | 53472 (0.46%) | | 53305 (2.25%) | | 5.78 | | 38722 | | 53 (28.19%) | | 28182 (52.87%) | | 1.374 |
| **Loratadine** | | 12 | | 53308 (0.46%) | | 53297 (2.25%) | | 5.73 | | 300 | | 5 (41.67%) | | 300 (0.56%) | | 1 |
| **Clonazepam** | | 208 | | 52313 (0.45%) | | 52242 (2.21%) | | 5.85 | | 52051 | | 64 (30.77%) | | 31717 (60.71%) | | 1.641 |
| **Tamsulosin** | | 89 | | 51375 (0.45%) | | 51262 (2.17%) | | 5.09 | | 16343 | | 30 (33.71%) | | 14591 (28.46%) | | 1.12 |
| **Clidinium / Chlordiazepoxide** | | 127 | | 51085 (0.44%) | | 51085 (2.16%) | | 5.29 | | 13279 | | 45 (35.43%) | | 11139 (21.80%) | | 1.192 |
| **Desloratadine** | | 5 | | 50773 (0.44%) | | 50762 (2.15%) | | 5.91 | | 175 | | 3 (60.00%) | | 174 (0.34%) | | 1.006 |
| **Alprazolam** | | 262 | | 50151 (0.44%) | | 50112 (2.12%) | | 6.09 | | 43401 | | 84 (32.06%) | | 27784 (55.44%) | | 1.562 |
| **Losartan Potassium / Hydrochlorothiazide** | | 221 | | 49821 (0.43%) | | 49821 (2.11%) | | 5.84 | | 65213 | | 68 (30.77%) | | 35891 (72.04%) | | 1.817 |
| **Sitagliptin / Metformin** | | 319 | | 46363 (0.40%) | | 46225 (1.95%) | | 5.8 | | 56850 | | 87 (27.27%) | | 30226 (65.39%) | | 1.881 |
| **Meloxicam** | | 261 | | 45857 (0.40%) | | 45846 (1.94%) | | 4.65 | | 40541 | | 75 (28.74%) | | 25577 (55.79%) | | 1.585 |
| **Calcium + Vitamin D3** | | 58 | | 45815 (0.40%) | | 45739 (1.93%) | | 5.65 | | 9803 | | 17 (29.31%) | | 8627 (18.86%) | | 1.136 |
| **Quetiapine** | | 357 | | 45580 (0.40%) | | 45060 (1.90%) | | 5.68 | | 94490 | | 113 (31.65%) | | 40262 (89.35%) | | 2.347 |
| **Cefalexin** | | 9 | | 41501 (0.36%) | | 41485 (1.75%) | | 3.99 | | 65 | | 2 (22.22%) | | 65 (0.16%) | | 1 |
| **Simethicone** | | 1 | | 40408 (0.35%) | | 39371 (1.66%) | | 5.63 | | 1290 | | 1 (100.00%) | | 1290 (3.28%) | | 1 |
| **Pregabalin** | | 154 | | 38405 (0.33%) | | 38206 (1.61%) | | 5.25 | | 16833 | | 43 (27.92%) | | 12908 (33.79%) | | 1.304 |
| **Carvedilol** | | 210 | | 37239 (0.32%) | | 37206 (1.57%) | | 7.3 | | 52800 | | 71 (33.81%) | | 28885 (77.64%) | | 1.828 |
| **Spironolactone** | | 142 | | 37098 (0.32%) | | 37080 (1.57%) | | 7.22 | | 46696 | | 42 (29.58%) | | 27445 (74.02%) | | 1.701 |
| **Allopurinol** | | 20 | | 36623 (0.32%) | | 36593 (1.55%) | | 7.07 | | 2694 | | 10 (50.00%) | | 2618 (7.15%) | | 1.029 |
| **Fluoxetine** | | 485 | | 33839 (0.29%) | | 32987 (1.39%) | | 5.23 | | 44950 | | 138 (28.45%) | | 24697 (74.87%) | | 1.82 |
| **Escitalopram** | | 436 | | 33437 (0.29%) | | 33379 (1.41%) | | 5.62 | | 43546 | | 123 (28.21%) | | 24472 (73.32%) | | 1.779 |
| **Nortriptyline** | | 312 | | 31300 (0.27%) | | 31217 (1.32%) | | 5.14 | | 31441 | | 91 (29.17%) | | 20865 (66.84%) | | 1.507 |
| **Empagliflozin / Linagliptin** | | 118 | | 29885 (0.26%) | | 29831 (1.26%) | | 6.27 | | 36049 | | 36 (30.51%) | | 22425 (75.17%) | | 1.608 |
| **Metoprolol Succinate** | | 184 | | 27322 (0.24%) | | 27273 (1.15%) | | 6.48 | | 30526 | | 57 (30.98%) | | 19486 (71.45%) | | 1.567 |
| **Risperidone** | | 230 | | 27229 (0.24%) | | 26880 (1.14%) | | 4.85 | | 32924 | | 76 (33.04%) | | 20587 (76.59%) | | 1.599 |
| **Baclofen** | | 79 | | 26979 (0.23%) | | 26920 (1.14%) | | 5.06 | | 13156 | | 30 (37.97%) | | 10902 (40.50%) | | 1.207 |
| **Acetaminophen/Caffeine/Ibuprofen** | | 270 | | 26427 (0.23%) | | 26427 (1.12%) | | 6.09 | | 25015 | | 71 (26.30%) | | 16040 (60.70%) | | 1.56 |
| **Domperidone** | | 240 | | 26245 (0.23%) | | 26244 (1.11%) | | 5.79 | | 21973 | | 85 (35.42%) | | 15775 (60.11%) | | 1.393 |
| **Biperiden** | | 35 | | 25166 (0.22%) | | 24585 (1.04%) | | 5.75 | | 12212 | | 13 (37.14%) | | 10794 (43.90%) | | 1.131 |
| **Citalopram** | | 427 | | 24587 (0.21%) | | 24531 (1.04%) | | 5.43 | | 31318 | | 116 (27.17%) | | 18055 (73.60%) | | 1.735 |
| **Ferrous Sulfate** | | 62 | | 24007 (0.21%) | | 23912 (1.01%) | | 5.98 | | 9670 | | 22 (35.48%) | | 8332 (34.84%) | | 1.161 |
| **Betahistine** | | 0 | | 23943 (0.21%) | | 23935 (1.01%) | | 5.08 | | 0 | | 0 (0.00%) | | 0 (0.00%) | | 0 |
| **Captopril** | | 191 | | 23195 (0.20%) | | 23172 (0.98%) | | 6.2 | | 39152 | | 54 (28.27%) | | 18977 (81.90%) | | 2.063 |
| **Bismuth Subcitrate** | | 1 | | 21434 (0.19%) | | 21433 (0.91%) | | 5.03 | | 12 | | 1 (100.00%) | | 12 (0.06%) | | 1 |
| **Calcitriol** | | 11 | | 20657 (0.18%) | | 20649 (0.87%) | | 6.96 | | 1528 | | 3 (27.27%) | | 1518 (7.35%) | | 1.007 |
| **Trifluoperazine** | | 200 | | 19066 (0.17%) | | 18737 (0.79%) | | 5 | | 16913 | | 71 (35.50%) | | 10845 (57.88%) | | 1.56 |
| **Finasteride** | | 1 | | 18985 (0.17%) | | 18978 (0.80%) | | 5.22 | | 0 | | 0 (0.00%) | | 0 (0.00%) | | 0 |
| **Carbamazepine** | | 655 | | 17025 (0.15%) | | 16887 (0.71%) | | 5.44 | | 44178 | | 165 (25.19%) | | 15655 (92.70%) | | 2.822 |
| **Hydroxychloroquine** | | 527 | | 16794 (0.15%) | | 16794 (0.71%) | | 5.83 | | 14948 | | 122 (23.15%) | | 10474 (62.37%) | | 1.427 |
| **Lansoprazole** | | 75 | | 16791 (0.15%) | | 16777 (0.71%) | | 5.58 | | 1553 | | 24 (32.00%) | | 1429 (8.52%) | | 1.087 |
| **Calcium Carbonate** | | 114 | | 16542 (0.14%) | | 16541 (0.70%) | | 5.84 | | 5474 | | 29 (25.44%) | | 4525 (27.36%) | | 1.21 |
| **Glibenclamide** | | 158 | | 16457 (0.14%) | | 16329 (0.69%) | | 6.03 | | 31038 | | 49 (31.01%) | | 14411 (88.25%) | | 2.154 |
| **Olanzapine** | | 423 | | 15995 (0.14%) | | 15968 (0.67%) | | 5.23 | | 42970 | | 126 (29.79%) | | 15248 (95.49%) | | 2.818 |
| **Mefenamic** | | 264 | | 15159 (0.13%) | | 15159 (0.64%) | | 4.5 | | 9452 | | 67 (25.38%) | | 6442 (42.50%) | | 1.467 |
| **Hematinic** | | 99 | | 15114 (0.13%) | | 7557 (0.32%) | | 5.84 | | 3341 | | 31 (31.31%) | | 2971 (39.31%) | | 1.125 |
| **Triamterene / Hydrochlorothiazide** | | 211 | | 15101 (0.13%) | | 15101 (0.64%) | | 6.38 | | 28551 | | 64 (30.33%) | | 12259 (81.18%) | | 2.329 |
| **Indomethacin** | | 238 | | 14943 (0.13%) | | 14861 (0.63%) | | 4.99 | | 16159 | | 72 (30.25%) | | 9400 (63.25%) | | 1.719 |
| **Diltiazem** | | 249 | | 14047 (0.12%) | | 14031 (0.59%) | | 6.86 | | 16645 | | 61 (24.50%) | | 9457 (67.40%) | | 1.76 |
| **Ursodeoxycholic Acid** | | 0 | | 13302 (0.12%) | | 13278 (0.56%) | | 4.85 | | 0 | | 0 (0.00%) | | 0 (0.00%) | | 0 |
| **Lithium** | | 298 | | 13024 (0.11%) | | 13024 (0.55%) | | 5.13 | | 18321 | | 81 (27.18%) | | 10576 (81.20%) | | 1.732 |
| **Mycophenolate Mofetil** | | 72 | | 11733 (0.10%) | | 11723 (0.50%) | | 5.58 | | 3175 | | 16 (22.22%) | | 2870 (24.48%) | | 1.106 |
| **Gemfibrozil** | | 55 | | 11036 (0.10%) | | 11035 (0.47%) | | 5.51 | | 4173 | | 16 (29.09%) | | 3804 (34.47%) | | 1.097 |
| **Atenolol** | | 156 | | 10353 (0.09%) | | 10349 (0.44%) | | 6.24 | | 11166 | | 45 (28.85%) | | 6625 (64.02%) | | 1.685 |
| **Mesalazine** | | 96 | | 9981 (0.09%) | | 8408 (0.36%) | | 4.37 | | 2582 | | 20 (20.83%) | | 2419 (28.77%) | | 1.067 |
| **Trihexyphenidyl** | | 51 | | 6655 (0.06%) | | 6650 (0.28%) | | 6.12 | | 5984 | | 17 (33.33%) | | 4349 (65.40%) | | 1.376 |
| **Vitamin D** | | 4 | | 6470 (0.06%) | | 6607 (0.28%) | | 6.05 | | 46 | | 3 (75.00%) | | 45 (0.68%) | | 1.022 |

Table 2. **The proportions of interactions found for the 100 most prescribed drugs (sorted in a descending order) in IHIO database.**

| **Name** | **Number of prescriptions (% of all prescriptions containing that target drug) with** | | | | | | | | | |
| --- | --- | --- | --- | --- | --- | --- | --- | --- | --- | --- |
|  | ***Moderate interactions*** | ***Major interactions*** | ***Contraindications*** | ***Only moderate interactions*** | ***Only major interactions*** | ***Only contraindications*** | ***Both moderate and major interactions*** | ***Both moderate interactions and contraindications*** | ***Both major interactions and contraindications*** | ***All types of interactions*** |
| **Acetaminophen** | 458 (0.11%) | 550 (0.13%) | 0 (0.00%) | 435 (0.10%) | 527 (0.12%) | 0 (0.00%) | 23 (0.01%) | 0 (0.00%) | 0 (0.00%) | 0 (0.00%) |
| **Atorvastatin** | 43165 (14.21%) | 28325 (9.33%) | 714 (0.24%) | 39443 (12.99%) | 24617 (8.11%) | 506 (0.17%) | 3597 (1.18%) | 97 (0.03%) | 83 (0.03%) | 28 (0.01%) |
| **Azithromycin** | 11782 (4.37%) | 48326 (17.93%) | 115 (0.04%) | 9186 (3.41%) | 45702 (16.96%) | 54 (0.02%) | 2571 (0.95%) | 8 (0.00%) | 36 (0.01%) | 17 (0.01%) |
| **Acetylsalicylic Acid** | 159935 (61.37%) | 177953 (68.29%) | 2002 (0.77%) | 45632 (17.51%) | 62816 (24.10%) | 311 (0.12%) | 113578 (43.58%) | 132 (0.05%) | 966 (0.37%) | 593 (0.23%) |
| **Adult Cold** | 8774 (3.51%) | 50127 (20.03%) | 21 (0.01%) | 7173 (2.87%) | 48524 (19.39%) | 13 (0.01%) | 1598 (0.64%) | 3 (0.00%) | 5 (0.00%) | 0 (0.00%) |
| **Vitamin B1** | 132 (0.06%) | 0 (0.00%) | 0 (0.00%) | 132 (0.06%) | 0 (0.00%) | 0 (0.00%) | 0 (0.00%) | 0 (0.00%) | 0 (0.00%) | 0 (0.00%) |
| **Pantoprazole** | 17316 (7.44%) | 6078 (2.61%) | 0 (0.00%) | 16584 (7.12%) | 5346 (2.30%) | 0 (0.00%) | 732 (0.31%) | 0 (0.00%) | 0 (0.00%) | 0 (0.00%) |
| **Metformin** | 116004 (52.58%) | 96986 (43.96%) | 0 (0.00%) | 59095 (26.78%) | 40077 (18.16%) | 0 (0.00%) | 56909 (25.79%) | 0 (0.00%) | 0 (0.00%) | 0 (0.00%) |
| **Losartan** | 51180 (24.32%) | 20244 (9.62%) | 0 (0.00%) | 43902 (20.87%) | 12966 (6.16%) | 0 (0.00%) | 7278 (3.46%) | 0 (0.00%) | 0 (0.00%) | 0 (0.00%) |
| **Famotidine** | 0 (0.00%) | 68034 (41.74%) | 52 (0.03%) | 0 (0.00%) | 68000 (41.71%) | 18 (0.01%) | 0 (0.00%) | 0 (0.00%) | 34 (0.02%) | 0 (0.00%) |
| **Ondansetron** | 346 (0.28%) | 62111 (50.38%) | 183 (0.15%) | 221 (0.18%) | 61869 (50.19%) | 63 (0.05%) | 123 (0.10%) | 1 (0.00%) | 118 (0.10%) | 1 (0.00%) |
| **Naproxen** | 11854 (7.74%) | 55204 (36.05%) | 46201 (30.17%) | 4307 (2.81%) | 34368 (22.45%) | 29314 (19.15%) | 5129 (3.35%) | 1180 (0.77%) | 14469 (9.45%) | 1238 (0.81%) |
| **Cefixime** | 0 (0.00%) | 270 (0.20%) | 0 (0.00%) | 0 (0.00%) | 270 (0.20%) | 0 (0.00%) | 0 (0.00%) | 0 (0.00%) | 0 (0.00%) | 0 (0.00%) |
| **Gabapentin** | 334 (0.27%) | 41707 (33.54%) | 0 (0.00%) | 86 (0.07%) | 41459 (33.34%) | 0 (0.00%) | 248 (0.20%) | 0 (0.00%) | 0 (0.00%) | 0 (0.00%) |
| **Montelukast** | 110 (0.09%) | 235 (0.19%) | 0 (0.00%) | 108 (0.09%) | 233 (0.19%) | 0 (0.00%) | 2 (0.00%) | 0 (0.00%) | 0 (0.00%) | 0 (0.00%) |
| **Valsartan** | 27481 (23.41%) | 12184 (10.38%) | 1615 (1.38%) | 21042 (17.92%) | 5914 (5.04%) | 1147 (0.98%) | 6097 (5.19%) | 295 (0.25%) | 126 (0.11%) | 47 (0.04%) |
| **Amlodipine** | 30300 (25.75%) | 15129 (12.86%) | 0 (0.00%) | 27239 (23.15%) | 12068 (10.26%) | 0 (0.00%) | 3061 (2.60%) | 0 (0.00%) | 0 (0.00%) | 0 (0.00%) |
| **Diclofenac** | 18291 (16.23%) | 47021 (41.73%) | 23011 (20.42%) | 7095 (6.30%) | 29462 (26.15%) | 13443 (11.93%) | 9066 (8.05%) | 1075 (0.95%) | 7438 (6.60%) | 1055 (0.94%) |
| **Rosuvastatin** | 634 (0.60%) | 5611 (5.27%) | 0 (0.00%) | 579 (0.54%) | 5556 (5.21%) | 0 (0.00%) | 55 (0.05%) | 0 (0.00%) | 0 (0.00%) | 0 (0.00%) |
| **Chlordiazepoxide** | 1332 (1.26%) | 19488 (18.46%) | 1 (0.00%) | 932 (0.88%) | 19088 (18.08%) | 1 (0.00%) | 400 (0.38%) | 0 (0.00%) | 0 (0.00%) | 0 (0.00%) |
| **Levothyroxine** | 52929 (50.34%) | 3569 (3.39%) | 0 (0.00%) | 50197 (47.74%) | 837 (0.80%) | 0 (0.00%) | 2732 (2.60%) | 0 (0.00%) | 0 (0.00%) | 0 (0.00%) |
| **Hydrochlorothiazide** | 35216 (34.71%) | 56078 (55.28%) | 0 (0.00%) | 12315 (12.14%) | 33177 (32.70%) | 0 (0.00%) | 22901 (22.57%) | 0 (0.00%) | 0 (0.00%) | 0 (0.00%) |
| **Ibuprofen** | 9043 (9.14%) | 32577 (32.92%) | 15826 (15.99%) | 3173 (3.21%) | 22166 (22.40%) | 9640 (9.74%) | 4742 (4.79%) | 517 (0.52%) | 5058 (5.11%) | 611 (0.62%) |
| **Metoprolol Tartrate** | 66218 (70.37%) | 7211 (7.66%) | 0 (0.00%) | 61686 (65.55%) | 2679 (2.85%) | 0 (0.00%) | 4532 (4.82%) | 0 (0.00%) | 0 (0.00%) | 0 (0.00%) |
| **Nitroglycerin** | 54678 (61.94%) | 14 (0.02%) | 63 (0.07%) | 54632 (61.89%) | 11 (0.01%) | 20 (0.02%) | 3 (0.00%) | 43 (0.05%) | 0 (0.00%) | 0 (0.00%) |
| **Folic Acid** | 765 (0.86%) | 14347 (16.06%) | 0 (0.00%) | 757 (0.85%) | 14339 (16.05%) | 0 (0.00%) | 8 (0.01%) | 0 (0.00%) | 0 (0.00%) | 0 (0.00%) |
| **Ciprofloxacin** | 10309 (11.94%) | 29904 (34.64%) | 53 (0.06%) | 5656 (6.55%) | 25230 (29.23%) | 17 (0.02%) | 4640 (5.38%) | 2 (0.00%) | 23 (0.03%) | 11 (0.01%) |
| **Metronidazole** | 127 (0.15%) | 39219 (45.13%) | 3 (0.00%) | 59 (0.07%) | 39150 (45.05%) | 1 (0.00%) | 67 (0.08%) | 0 (0.00%) | 1 (0.00%) | 1 (0.00%) |
| **Empagliflozin** | 51480 (61.19%) | 27905 (33.17%) | 0 (0.00%) | 34936 (41.53%) | 11361 (13.50%) | 0 (0.00%) | 16544 (19.67%) | 0 (0.00%) | 0 (0.00%) | 0 (0.00%) |
| **Cetirizine** | 8 (0.01%) | 33808 (40.33%) | 0 (0.00%) | 7 (0.01%) | 33807 (40.33%) | 0 (0.00%) | 1 (0.00%) | 0 (0.00%) | 0 (0.00%) | 0 (0.00%) |
| **Co-amoxiclav** | 0 (0.00%) | 735 (0.88%) | 0 (0.00%) | 0 (0.00%) | 735 (0.88%) | 0 (0.00%) | 0 (0.00%) | 0 (0.00%) | 0 (0.00%) | 0 (0.00%) |
| **Amoxicillin** | 0 (0.00%) | 577 (0.72%) | 0 (0.00%) | 0 (0.00%) | 577 (0.72%) | 0 (0.00%) | 0 (0.00%) | 0 (0.00%) | 0 (0.00%) | 0 (0.00%) |
| **Propranolol** | 42757 (54.60%) | 15535 (19.84%) | 383 (0.49%) | 36650 (46.80%) | 9503 (12.14%) | 151 (0.19%) | 5936 (7.58%) | 136 (0.17%) | 61 (0.08%) | 35 (0.04%) |
| **Prednisolone** | 7198 (9.27%) | 19673 (25.35%) | 81 (0.10%) | 4838 (6.23%) | 17329 (22.33%) | 35 (0.05%) | 2325 (3.00%) | 27 (0.03%) | 11 (0.01%) | 8 (0.01%) |
| **Hyoscine** | 0 (0.00%) | 23417 (38.59%) | 34 (0.06%) | 0 (0.00%) | 23409 (38.58%) | 26 (0.04%) | 0 (0.00%) | 0 (0.00%) | 8 (0.01%) | 0 (0.00%) |
| **Celecoxib** | 8033 (11.04%) | 33003 (45.35%) | 13320 (18.30%) | 6614 (9.09%) | 21674 (29.78%) | 4883 (6.71%) | 5994 (8.24%) | 260 (0.36%) | 2438 (3.35%) | 452 (0.62%) |
| **Omeprazole** | 9840 (13.86%) | 6906 (9.73%) | 0 (0.00%) | 8554 (12.05%) | 5620 (7.92%) | 0 (0.00%) | 1286 (1.81%) | 0 (0.00%) | 0 (0.00%) | 0 (0.00%) |
| **Sertraline** | 15302 (21.76%) | 48514 (68.99%) | 551 (0.78%) | 4999 (7.11%) | 37925 (53.93%) | 128 (0.18%) | 10192 (14.49%) | 26 (0.04%) | 312 (0.44%) | 85 (0.12%) |
| **Gliclazide** | 25870 (38.57%) | 2489 (3.71%) | 0 (0.00%) | 24783 (36.95%) | 1402 (2.09%) | 0 (0.00%) | 1087 (1.62%) | 0 (0.00%) | 0 (0.00%) | 0 (0.00%) |
| **Bisoprolol Fumarate** | 43995 (66.21%) | 2200 (3.31%) | 0 (0.00%) | 42606 (64.12%) | 811 (1.22%) | 0 (0.00%) | 1389 (2.09%) | 0 (0.00%) | 0 (0.00%) | 0 (0.00%) |
| **Acetaminophen / Codeine** | 74 (0.11%) | 48418 (74.90%) | 14 (0.02%) | 17 (0.03%) | 48352 (74.80%) | 5 (0.01%) | 57 (0.09%) | 0 (0.00%) | 9 (0.01%) | 0 (0.00%) |
| **Furosemide** | 9579 (17.42%) | 42339 (76.99%) | 3 (0.01%) | 1783 (3.24%) | 34543 (62.82%) | 3 (0.01%) | 7796 (14.18%) | 0 (0.00%) | 0 (0.00%) | 0 (0.00%) |
| **Clopidogrel** | 31279 (52.34%) | 48054 (80.41%) | 0 (0.00%) | 4142 (6.93%) | 20917 (35.00%) | 0 (0.00%) | 27137 (45.41%) | 0 (0.00%) | 0 (0.00%) | 0 (0.00%) |
| **Acetylcysteine** | 1107 (1.86%) | 0 (0.00%) | 0 (0.00%) | 0 (0.00%) | 1107 (1.86%) | 0 (0.00%) | 0 (0.00%) | 0 (0.00%) | 0 (0.00%) | 0 (0.00%) |
| **Valproate** | 21047 (37.14%) | 21407 (37.78%) | 0 (0.00%) | 13770 (24.30%) | 14130 (24.94%) | 0 (0.00%) | 7277 (12.84%) | 0 (0.00%) | 0 (0.00%) | 0 (0.00%) |
| **Amlodipine / Valsartan** | 21399 (40.14%) | 9960 (18.68%) | 896 (1.68%) | 17475 (32.78%) | 6163 (11.56%) | 526 (0.99%) | 3648 (6.84%) | 221 (0.41%) | 94 (0.18%) | 55 (0.10%) |
| **Loratadine** | 0 (0.00%) | 300 (0.56%) | 0 (0.00%) | 0 (0.00%) | 300 (0.56%) | 0 (0.00%) | 0 (0.00%) | 0 (0.00%) | 0 (0.00%) | 0 (0.00%) |
| **Clonazepam** | 831 (1.59%) | 31422 (60.15%) | 0 (0.00%) | 295 (0.56%) | 30886 (59.12%) | 0 (0.00%) | 536 (1.03%) | 0 (0.00%) | 0 (0.00%) | 0 (0.00%) |
| **Tamsulosin** | 8774 (17.12%) | 6937 (13.53%) | 0 (0.00%) | 7654 (14.93%) | 5817 (11.35%) | 0 (0.00%) | 1120 (2.18%) | 0 (0.00%) | 0 (0.00%) | 0 (0.00%) |
| **Clidinium / Chlordiazepoxide** | 570 (1.12%) | 10782 (21.11%) | 10 (0.02%) | 350 (0.69%) | 10559 (20.67%) | 7 (0.01%) | 220 (0.43%) | 0 (0.00%) | 3 (0.01%) | 0 (0.00%) |
| **Desloratadine** | 0 (0.00%) | 174 (0.34%) | 0 (0.00%) | 0 (0.00%) | 174 (0.34%) | 0 (0.00%) | 0 (0.00%) | 0 (0.00%) | 0 (0.00%) | 0 (0.00%) |
| **Alprazolam** | 11716 (23.38%) | 21888 (43.68%) | 64 (0.13%) | 5862 (11.70%) | 16027 (31.98%) | 23 (0.05%) | 5831 (11.64%) | 11 (0.02%) | 18 (0.04%) | 12 (0.02%) |
| **Losartan Potassium / Hydrochlorothiazide** | 18587 (37.31%) | 30235 (60.69%) | 0 (0.00%) | 5656 (11.35%) | 17304 (34.73%) | 0 (0.00%) | 12931 (25.95%) | 0 (0.00%) | 0 (0.00%) | 0 (0.00%) |
| **Sitagliptin / Metformin** | 19808 (42.85%) | 21675 (46.89%) | 0 (0.00%) | 8551 (18.50%) | 10418 (22.54%) | 0 (0.00%) | 11257 (24.35%) | 0 (0.00%) | 0 (0.00%) | 0 (0.00%) |
| **Meloxicam** | 4406 (9.61%) | 20275 (44.22%) | 7366 (16.07%) | 1375 (3.00%) | 14191 (30.95%) | 3762 (8.21%) | 2645 (5.77%) | 165 (0.36%) | 3218 (7.02%) | 221 (0.48%) |
| **Calcium + Vitamin D3** | 8554 (18.70%) | 130 (0.28%) | 0 (0.00%) | 8497 (18.58%) | 73 (0.16%) | 0 (0.00%) | 57 (0.12%) | 0 (0.00%) | 0 (0.00%) | 0 (0.00%) |
| **Quetiapine** | 1128 (2.50%) | 40024 (88.82%) | 451 (1.00%) | 204 (0.45%) | 38723 (85.94%) | 31 (0.07%) | 884 (1.96%) | 3 (0.01%) | 380 (0.84%) | 37 (0.08%) |
| **Cefalexin** | 0 (0.00%) | 65 (0.16%) | 0 (0.00%) | 0 (0.00%) | 65 (0.16%) | 0 (0.00%) | 0 (0.00%) | 0 (0.00%) | 0 (0.00%) | 0 (0.00%) |
| **Simethicone** | 1290 (3.28%) | 0 (0.00%) | 0 (0.00%) | 1290 (3.28%) | 0 (0.00%) | 0 (0.00%) | 0 (0.00%) | 0 (0.00%) | 0 (0.00%) | 0 (0.00%) |
| **Pregabalin** | 0 (0.00%) | 12908 (33.79%) | 0 (0.00%) | 0 (0.00%) | 12908 (33.79%) | 0 (0.00%) | 0 (0.00%) | 0 (0.00%) | 0 (0.00%) | 0 (0.00%) |
| **Carvedilol** | 27300 (73.38%) | 5287 (14.21%) | 87 (0.23%) | 3719 (10.00%) | 23518 (63.21%) | 59 (0.16%) | 1561 (4.20%) | 3 (0.01%) | 21 (0.06%) | 4 (0.01%) |
| **Spironolactone** | 19448 (52.45%) | 18706 (50.45%) | 472 (1.27%) | 8537 (23.02%) | 7774 (20.97%) | 95 (0.26%) | 10662 (28.75%) | 107 (0.29%) | 128 (0.35%) | 142 (0.38%) |
| **Allopurinol** | 241 (0.66%) | 2387 (6.52%) | 0 (0.00%) | 231 (0.63%) | 2377 (6.50%) | 0 (0.00%) | 10 (0.03%) | 0 (0.00%) | 0 (0.00%) | 0 (0.00%) |
| **Fluoxetine** | 5056 (15.33%) | 22980 (69.66%) | 139 (0.42%) | 1684 (5.11%) | 19507 (59.14%) | 31 (0.09%) | 3367 (10.21%) | 2 (0.01%) | 103 (0.31%) | 3 (0.01%) |
| **Escitalopram** | 768 (2.30%) | 24254 (72.66%) | 154 (0.46%) | 170 (0.51%) | 23556 (70.57%) | 45 (0.13%) | 592 (1.77%) | 3 (0.01%) | 103 (0.31%) | 3 (0.01%) |
| **Nortriptyline** | 173 (0.55%) | 20771 (66.54%) | 206 (0.66%) | 39 (0.12%) | 20486 (65.62%) | 55 (0.18%) | 134 (0.43%) | 0 (0.00%) | 151 (0.48%) | 0 (0.00%) |
| **Empagliflozin / Linagliptin** | 17883 (59.95%) | 9785 (32.80%) | 0 (0.00%) | 12640 (42.37%) | 4542 (15.23%) | 0 (0.00%) | 5243 (17.58%) | 0 (0.00%) | 0 (0.00%) | 0 (0.00%) |
| **Metoprolol Succinate** | 18651 (68.39%) | 2052 (7.52%) | 0 (0.00%) | 17434 (63.92%) | 835 (3.06%) | 0 (0.00%) | 1217 (4.46%) | 0 (0.00%) | 0 (0.00%) | 0 (0.00%) |
| **Risperidone** | 1801 (6.70%) | 20017 (74.47%) | 318 (1.18%) | 488 (1.82%) | 18555 (69.03%) | 56 (0.21%) | 1226 (4.56%) | 26 (0.10%) | 175 (0.65%) | 61 (0.23%) |
| **Baclofen** | 555 (2.06%) | 10637 (39.51%) | 0 (0.00%) | 290 (1.08%) | 10347 (38.44%) | 0 (0.00%) | 265 (0.98%) | 0 (0.00%) | 0 (0.00%) | 0 (0.00%) |
| **Acetaminophen/Caffeine/Ibuprofen** | 3823 (14.47%) | 11067 (41.88%) | 6412 (24.26%) | 3436 (13.00%) | 6454 (24.42%) | 1932 (7.31%) | 2327 (8.81%) | 295 (1.12%) | 1242 (4.70%) | 354 (1.34%) |
| **Domperidone** | 16 (0.06%) | 15426 (58.78%) | 526 (2.00%) | 10 (0.04%) | 15233 (58.04%) | 339 (1.29%) | 6 (0.02%) | 0 (0.00%) | 187 (0.71%) | 0 (0.00%) |
| **Biperiden** | 0 (0.00%) | 10791 (43.89%) | 4 (0.02%) | 0 (0.00%) | 10790 (43.89%) | 3 (0.01%) | 0 (0.00%) | 0 (0.00%) | 1 (0.00%) | 0 (0.00%) |
| **Citalopram** | 22 (0.09%) | 18020 (73.46%) | 150 (0.61%) | 5 (0.02%) | 17883 (72.90%) | 30 (0.12%) | 17 (0.07%) | 0 (0.00%) | 120 (0.49%) | 0 (0.00%) |
| **Ferrous Sulfate** | 8254 (34.52%) | 123 (0.51%) | 0 (0.00%) | 8209 (34.33%) | 78 (0.33%) | 0 (0.00%) | 45 (0.19%) | 0 (0.00%) | 0 (0.00%) | 0 (0.00%) |
| **Betahistine** | 0 (0.00%) | 0 (0.00%) | 0 (0.00%) | 0 (0.00%) | 0 (0.00%) | 0 (0.00%) | 0 (0.00%) | 0 (0.00%) | 0 (0.00%) | 0 (0.00%) |
| **Captopril** | 9644 (41.62%) | 14729 (63.56%) | 1449 (6.25%) | 3423 (14.77%) | 8539 (36.85%) | 480 (2.07%) | 5566 (24.02%) | 345 (1.49%) | 314 (1.36%) | 310 (1.34%) |
| **Bismuth Subcitrate** | 0 (0.00%) | 12 (0.06%) | 0 (0.00%) | 0 (0.00%) | 12 (0.06%) | 0 (0.00%) | 0 (0.00%) | 0 (0.00%) | 0 (0.00%) | 0 (0.00%) |
| **Calcitriol** | 1518 (7.35%) | 0 (0.00%) | 0 (0.00%) | 1518 (7.35%) | 0 (0.00%) | 0 (0.00%) | 0 (0.00%) | 0 (0.00%) | 0 (0.00%) | 0 (0.00%) |
| **Trifluoperazine** | 1571 (8.38%) | 9998 (53.36%) | 604 (3.22%) | 577 (3.08%) | 8702 (46.44%) | 262 (1.40%) | 962 (5.13%) | 8 (0.04%) | 310 (1.65%) | 24 (0.13%) |
| **Finasteride** | 0 (0.00%) | 0 (0.00%) | 0 (0.00%) | 0 (0.00%) | 0 (0.00%) | 0 (0.00%) | 0 (0.00%) | 0 (0.00%) | 0 (0.00%) | 0 (0.00%) |
| **Carbamazepine** | 6121 (36.25%) | 14970 (88.65%) | 32 (0.19%) | 682 (4.04%) | 9515 (56.35%) | 3 (0.02%) | 5426 (32.13%) | 0 (0.00%) | 16 (0.09%) | 13 (0.08%) |
| **Hydroxychloroquine** | 0 (0.00%) | 10474 (62.37%) | 1 (0.01%) | 0 (0.00%) | 10473 (62.36%) | 0 (0.00%) | 0 (0.00%) | 0 (0.00%) | 1 (0.01%) | 0 (0.00%) |
| **Lansoprazole** | 1145 (6.82%) | 333 (1.98%) | 0 (0.00%) | 1096 (6.53%) | 284 (1.69%) | 0 (0.00%) | 49 (0.29%) | 0 (0.00%) | 0 (0.00%) | 0 (0.00%) |
| **Calcium Carbonate** | 4038 (24.41%) | 638 (3.86%) | 0 (0.00%) | 3887 (23.50%) | 487 (2.94%) | 0 (0.00%) | 151 (0.91%) | 0 (0.00%) | 0 (0.00%) | 0 (0.00%) |
| **Glibenclamide** | 6800 (41.64%) | 13413 (82.14%) | 0 (0.00%) | 998 (6.11%) | 7611 (46.61%) | 0 (0.00%) | 5802 (35.53%) | 0 (0.00%) | 0 (0.00%) | 0 (0.00%) |
| **Olanzapine** | 5277 (33.05%) | 14651 (91.75%) | 220 (1.38%) | 592 (3.71%) | 9860 (61.75%) | 5 (0.03%) | 4576 (28.66%) | 0 (0.00%) | 106 (0.66%) | 109 (0.68%) |
| **Mefenamic** | 1177 (7.76%) | 4805 (31.70%) | 1774 (11.70%) | 419 (2.76%) | 3595 (23.72%) | 1173 (7.74%) | 654 (4.31%) | 45 (0.30%) | 497 (3.28%) | 59 (0.39%) |
| **Hematinic** | 2515 (33.28%) | 586 (7.75%) | 0 (0.00%) | 2385 (31.56%) | 456 (6.03%) | 0 (0.00%) | 130 (1.72%) | 0 (0.00%) | 0 (0.00%) | 0 (0.00%) |
| **Triamterene / Hydrochlorothiazide** | 9589 (63.50%) | 10079 (66.74%) | 344 (2.28%) | 2117 (14.02%) | 2545 (16.85%) | 40 (0.26%) | 7253 (48.03%) | 23 (0.15%) | 85 (0.56%) | 196 (1.30%) |
| **Indomethacin** | 2262 (15.22%) | 7614 (51.23%) | 2749 (18.50%) | 481 (3.24%) | 4582 (30.83%) | 1261 (8.49%) | 1588 (10.69%) | 44 (0.30%) | 1295 (8.71%) | 149 (1.00%) |
| **Diltiazem** | 3372 (24.03%) | 8523 (60.74%) | 57 (0.41%) | 905 (6.45%) | 6040 (43.05%) | 25 (0.18%) | 2455 (17.50%) | 4 (0.03%) | 20 (0.14%) | 8 (0.06%) |
| **Ursodeoxycholic Acid** | 0 (0.00%) | 0 (0.00%) | 0 (0.00%) | 0 (0.00%) | 0 (0.00%) | 0 (0.00%) | 0 (0.00%) | 0 (0.00%) | 0 (0.00%) | 0 (0.00%) |
| **Lithium** | 1354 (10.40%) | 10312 (79.18%) | 0 (0.00%) | 264 (2.03%) | 9222 (70.81%) | 0 (0.00%) | 1090 (8.37%) | 0 (0.00%) | 0 (0.00%) | 0 (0.00%) |
| **Mycophenolate Mofetil** | 137 (1.17%) | 2791 (23.81%) | 0 (0.00%) | 79 (0.67%) | 2733 (23.31%) | 0 (0.00%) | 58 (0.49%) | 0 (0.00%) | 0 (0.00%) | 0 (0.00%) |
| **Gemfibrozil** | 625 (5.66%) | 3307 (29.97%) | 134 (1.21%) | 417 (3.78%) | 3071 (27.83%) | 58 (0.53%) | 182 (1.65%) | 22 (0.20%) | 50 (0.45%) | 4 (0.04%) |
| **Atenolol** | 6557 (63.36%) | 223 (2.15%) | 0 (0.00%) | 6402 (61.86%) | 68 (0.66%) | 0 (0.00%) | 155 (1.50%) | 0 (0.00%) | 0 (0.00%) | 0 (0.00%) |
| **Mesalazine** | 303 (3.60%) | 2169 (25.80%) | 0 (0.00%) | 250 (2.97%) | 2116 (25.17%) | 0 (0.00%) | 53 (0.63%) | 0 (0.00%) | 0 (0.00%) | 0 (0.00%) |
| **Trihexyphenidyl** | 8774 (131.94%) | 2477 (37.25%) | 1 (0.02%) | 1871 (28.14%) | 1529 (22.99%) | 1 (0.02%) | 948 (14.26%) | 0 (0.00%) | 0 (0.00%) | 0 (0.00%) |
| **Vitamin D** | 45 (0.68%) | 0 (0.00%) | 0 (0.00%) | 45 (0.68%) | 0 (0.00%) | 0 (0.00%) | 0 (0.00%) | 0 (0.00%) | 0 (0.00%) | 0 (0.00%) |
