# Supplementary material for: Prevalence, predictors, and clinical relevance of drug–drug interactions in outpatient prescribing: A national cross-sectional study
Source: PLoS One. 2026 Apr 8;21(4):e0345076. doi: 10.1371/journal.pone.0345076 (PMC13061183; doi:10.1371/journal.pone.0345076)
Supplement: S2 Table — This table presents the three most frequent drug interaction partners for each of the 100 most commonly prescribed medications in the IHIO database, categorized by severity level (contraindicated, major, and moderate). Numbers represent the absolute frequency of co-prescription, while percentages (in parentheses) indicate the proportion relative to all prescriptions containing the target drug listed in the first column. Empty cells indicate no interactions were identified in that severity category. (DOCX) [file pone.0345076.s002.DOCX]

**S2 Table 1.** Top Three Interacting Drugs for Each of the 100 Most Frequently Prescribed Medications, Stratified by Interaction Severity

| **Drug name** | **Contraindicated** | **Major Interactions** | **Moderate Interactions** |
| --- | --- | --- | --- |
| ACETAMINOPHEN |  | Carbamazepine: 541 (0.12%),  Imatinib: 8 (0.00%),  Isoniazid: 1 (0.00%) | Warfarin: 230 (0.05%),  Phenytoin: 225 (0.05%),  Ethinyl estradiol: 5 (0.00%) |
| ACETAMINOPHEN / CODEINE | Linezolid: 13 (0.02%),  Selegiline: 1 (0.00%) | Diphenhydramine: 15325 (23.69%),  Adult cold: 13437 (20.77%),  Dexamethasone: 8428 (13.03%) | Warfarin: 51 (0.08%),  Clonidine: 19 (0.03%),  Ginseng: 3 (0.00%) |
| ACETAMINOPHEN/CAFFEINE/IBUPROFEN | Ketorolac: 6412 (24.26%) | Dexamethasone: 3190 (12.07%),  Diclofenac: 1565 (5.92%),  Piroxicam: 1533 (5.80%) | Losartan: 1320 (4.99%),  Propranolol: 1040 (3.94%),  Levofloxacin: 683 (2.58%) |
| ACETYLCYSTEINE |  |  | Nitroglycerin: 1045 (1.75%),  Carbamazepine: 79 (0.13%) |
| ACETYLSALICYLIC ACID | Ketorolac: 2002 (0.77%) | Metformin: 61401 (23.55%),  Clopidogrel: 37956 (14.56%), hydrochlorothiazide: 35357 (13.56%) | Metoprolol: 58676 (22.50%),  Nitroglycerin: 54210 (20.79%),  Bisoprolol: 33023 (12.66%) |
| ADULT COLD | Potassium citrate: 15 (0.01%),  Linezolid: 2 (0.00%),  Selegiline: 2 (0.00%) | Codeine: 21665 (8.63%),  Dextromethorphan: 15905 (6.34%), ondansetron: 10534 (4.20%) | Metformin: 7365 (2.93%),  Insulin: 1748 (0.70%),  Warfarin: 123 (0.05%) |
| ALLOPURINOL |  | Enalapril: 796 (2.17%),  Warfarin: 726 (1.98%),  Captopril: 671 (1.83%) | Theophylline: 241 (0.66%) |
| ALPRAZOLAM | Clarithromycin: 46 (0.09%), itraconazole: 16 (0.03%),  Voriconazole: 2 (0.00%) | Gabapentin: 3428 (6.84%),  Chlordiazepoxide: 3239 (6.46%),  Quetiapine: 2761 (5.51%) | Sertraline: 5658 (11.28%),  Omeprazole: 3196 (6.37%),  Fluoxetine: 2214 (4.41%) |
| AMLODIPINE |  | Clopidogrel: 10923 (9.23%),  Tacrolimus: 996 (0.84%),  Warfarin: 948 (0.80%) | Metformin: 28225 (23.86%),  Ciprofloxacin: 1226 (1.04%),  Diltiazem: 872 (0.74%) |
| AMLODIPINE / VALSARTAN | Captopril: 803 (1.80%),  Enalapril: 91 (0.20%),  Lisinopril: 4 (0.01%) | Clopidogrel: 6028 (13.49%),  Furosemide: 3116 (6.97%),  Warfarin: 366 (0.82%) | Metformin: 12993 (29.08%),  Insulin Aspart: 2186 (4.89%),  Spironolactone: 1675 (3.75%) |
| AMOXICILLIN |  | Tetracycline: 162 (0.20%),  Warfarin: 82 (0.10%),  Rivaroxaban: 77 (0.10%) |  |
| ATENOLOL |  | Diltiazem: 133 (1.28%),  Salmeterol: 24 (0.23%),  Formoterol: 20 (0.19%) | Acetylsalicylic acid: 3479 (33.60%),  Metformin: 2916 (28.17%),  Empagliflozin: 571 (5.52%) |
| ATORVASTATIN | Colchicine: 713 (0.23%),  Posaconazole: 1 (0.00%) | Diltiazem: 5307 (1.74%),  Ciprofloxacin: 4702 (1.54%),  Warfarin: 4028 (1.32%) | Clopidogrel: 30572 (10.04%),  Pioglitazone: 4668 (1.53%),  Azithromycin: 4540 (1.49%) |
| AZITHROMYCIN | Colchicine: 88 (0.03%),  Thioridazine: 23 (0.01%),  Pimozide: 4 (0.00%) | Famotidine: 23353 (8.64%),  Ondansetron: 12454 (4.61%),  Metronidazole: 5512 (2.04%) | Theophylline: 7278 (2.69%),  Atorvastatin: 4540 (1.68%),  Carbamazepine: 159 (0.06%) |
| BACLOFEN |  | Gabapentin: 6758 (25.05%),  Pregabalin: 1565 (5.80%),  Codeine: 862 (3.20%) | Tizanidine: 495 (1.83%),  Clonidine: 62 (0.23%) |
| BETAHISTINE |  |  |  |
| BIPERIDEN | Potassium chloride: 4 (0.02%) | Quetiapine: 7036 (27.96%),  Olanzapine: 2979 (11.84%),  Clozapine: 1424 (5.66%) |  |
| BISMUTH SUBCITRATE |  | Digoxin: 12 (0.06%) |  |
| BISOPROLOL FUMARATE |  | Salmeterol: 1048 (1.57%),  Diltiazem: 621 (0.93%),  Formoterol: 322 (0.48%) | Acetylsalicylic acid: 33023 (49.62%),  Metformin: 10300 (15.48%),  Empagliflozin: 8068 (12.12%) |
| CALCITRIOL |  |  | Hydrochlorothiazide: 1315 (6.37%),  Magnesium: 210 (1.02%),  Metolazone: 3 (0.01%) |
| CALCIUM CARBONATE |  | Hydroxychloroquine: 429 (2.59%),  Sodium polystyrene sulfonate: 69 (0.42%),  Mefenamic acid: 63 (0.38%) | Acetylsalicylic acid: 1960 (11.85%),  Levothyroxine: 1382 (8.35%),  Hydrochlorothiazide: 462 (2.79%) |
| CALCIUM+VITAMIN D3 |  | Digoxin: 128 (0.28%),  Sodium polystyrene sulfonate: 2 (0.00%) | Acetylsalicylic acid: 5499 (12.00%),  Hydrochlorothiazide: 1935 (4.22%),  Ciprofloxacin: 933 (2.04%) |
| CAPTOPRIL | Valsartan: 1403 (6.05%)  Colchicine: 48 (0.21%),  Sacubitril: 11 (0.05%), | Acetylsalicylic acid: 9468 (40.82%), furosemide: 3957 (17.06%),  Potassium: 2640 (11.38%) | Metformin: 3952 (17.04%),  Hydrochlorothiazide: 2177 (9.39%),  Empagliflozin: 1668 (7.19%) |
| CARBAMAZEPINE | Ranolazine: 13 (0.08%),  Selegiline: 6 (0.04%),  Linezolid: 6 (0.04%),  Procarbazine: 4 (0.02%) | Valproate: 3858 (22.66%),  Clonazepam: 2658 (15.61%),  Quetiapine: 2557 (15.02%) | Propranolol: 2081 (12.22%),  Levetiracetam: 1556 (9.14%),  Lithium: 1110 (6.52%) |
| CARVEDILOL | Colchicine: 87 (0.23%) | Digoxin: 1335 (3.58%),  Sertraline: 1243 (3.34%),  Amiodarone: 495 (1.33%) | Acetylsalicylic acid: 19781 (53.12%),  Metformin: 7951 (21.35%),  Empagliflozin: 5362 (14.40%) |
| CEFALEXIN |  | Warfarin: 43 (0.10%),  Methotrexate: 22 (0.05%) |  |
| CEFIXIME |  | Warfarin: 110 (0.08%),  Methotrexate: 90 (0.07%),  Estradiol: 55 (0.04%) |  |
| CELECOXIB | Ketorolac: 13320 (18.29%) | Methylprednisolone: 7897 (10.84%), piroxicam: 4563 (6.27%),  Acetylsalicylic acid: 2822 (3.88%) | Losartan: 3066 (4.21%),  Propranolol: 1290 (1.77%),  Metoprolol: 1217 (1.67%) |
| CETIRIZINE |  | Diphenhydramine: 26503 (31.59%),  Codeine: 5883 (7.01%),  Chlordiazepoxide: 1346 (1.60%) | Clonidine: 8 (0.01%) |
| CHLORDIAZEPOXIDE | Flumazenil: 1 (0.00%) | Gabapentin: 4493 (4.22%),  Alprazolam: 3239 (3.04%),  Clonazepam: 2352 (2.21%) | Theophylline: 702 (0.66%),  Warfarin: 298 (0.28%),  Tizanidine: 168 (0.16%) |
| CIPROFLOXACIN | Tizanidine: 51 (0.06%),  Thioridazine: 1 (0.00%),  Ziprasidone: 1 (0.00%) | Metronidazole: 9080 (10.28%),  atorvastatin: 4702 (5.32%),  Ondansetron: 4270 (4.83%) | Diclofenac: 4581 (5.19%),  Levothyroxine: 1496 (1.69%),  Calcium: 1458 (1.65%) |
| CITALOPRAM | Thioridazine: 49 (0.20%),  Metoclopramide: 39 (0.16%),  Pimozide: 25 (0.10%) | Acetylsalicylic acid: 3722 (15.14%),  Quetiapine: 3014 (12.26%),  Nortriptyline: 1661 (6.76%) | Ginkgo: 22 (0.09%) |
| CLIDINIUM / CHLORDIAZEPOXIDE | Potassium chloride: 2 (0.00%), potassium citrate: 8 (0.02%) | Hyoscine: 3474 (6.80%),  Gabapentin: 1476 (2.89%),  Alprazolam: 1309 (2.56%) | Theophylline: 429 (0.84%),  Warfarin: 60 (0.12%),  Tizanidine: 49 (0.10%) |
| CLONAZEPAM |  | Quetiapine: 9169 (17.53%),  Gabapentin: 5120 (9.79%),  Olanzapine: 3129 (5.98%) | Clonidine: 397 (0.76%),  Theophylline: 253 (0.48%),  Amiodarone: 78 (0.15%) |
| CLOPIDOGREL |  | Acetylsalicylic acid: 37956 (63.51%), amlodipine: 10923 (18.28%),  Sertraline: 2065 (3.46%) | Atorvastatin: 30572 (51.16%),  Lansoprazole: 417 (0.70%),  Pioglitazone: 270 (0.45%) |
| CO-AMOXICLAV |  | Apixaban: 204 (0.25%),  Rivaroxaban: 140 (0.17%),  Doxycycline: 124 (0.15%) |  |
| DESLORATADINE |  | Clopidogrel: 108 (0.21%),  Gemfibrozil: 40 (0.08%),  Carbamazepine: 27 (0.05%) |  |
| DICLOFENAC | Ketorolac: 23011 (19.53%) | Piroxicam: 8513 (7.22%),  Dexamethasone: 7522 (6.38%), methylprednisolone: 6795 (5.77%) | Losartan: 6376 (5.41%),  Ciprofloxacin: 4581 (3.89%),  Metoprolol: 2399 (2.04%) |
| DILTIAZEM | Colchicine: 57 (0.41%) | Atorvastatin: 5307 (37.78%),  Clopidogrel: 1505 (10.71%),  Metoprolol: 1361 (9.69%) | Metformin: 2310 (16.44%),  Amlodipine: 872 (6.21%),  Theophylline: 193 (1.37%) |
| DOMPERIDONE | Clarithromycin: 449 (1.71%), fluconazole: 73 (0.28%),  Ketoconazole: 4 (0.02%), | Famotidine: 5642 (21.50%),  Ondansetron: 3790 (14.44%),  Metronidazole: 2194 (8.36%) | Cabergoline: 9 (0.03%),  Bromocriptine: 7 (0.03%) |
| EMPAGLIFLOZIN |  | Insulin: 14041 (16.56%),  Hydrochlorothiazide: 7637 (9.01%), furosemide: 6712 (7.92%) | Gliclazide: 23656 (27.90%),  Metoprolol: 10652 (12.56%),  Levothyroxine: 9723 (11.47%) |
| EMPAGLIFLOZIN / LINAGLIPTIN |  | Insulin Aspart: 3590 (12.01%),  Insulin: 3293 (11.02%),  Hydrochlorothiazide: 2834 (9.48%) | Gliclazide: 10006 (33.48%),  Levothyroxine: 3460 (11.58%),  Metoprolol: 3389 (11.34%) |
| ESCITALOPRAM | Fluconazole: 30 (0.09%),  Linezolid: 8 (0.02%),  Metoclopramide: 39 (0.12%),  Pimozide: 60 (0.18%),  Selegiline: 18 (0.05%) | Propranolol: 4665 (13.95%),  Acetylsalicylic acid: 3920 (11.72%), quetiapine: 3705 (11.08%) | Lamotrigine: 694 (2.08%),  Ginkgo: 74 (0.22%) |
| FAMOTIDINE | Thioridazine: 40 (0.02%),  Pimozide: 12 (0.01%) | Azithromycin: 23353 (14.25%),  Ondansetron: 22501 (13.73%),  Metronidazole: 7431 (4.54%) |  |
| FERROUS SULFATE |  | Levodopa: 114 (0.47%),  Carbidopa: 63 (0.26%),  Ethinyl estradiol: 9 (0.04%) | Pantoprazole: 3327 (13.86%),  Levothyroxine: 2515 (10.48%),  Omeprazole: 1037 (4.32%) |
| FINASTERIDE |  |  |  |
| FLUOXETINE | Thioridazine: 75 (0.22%),  Pimozide: 56 (0.17%),  Selegiline: 7 (0.02%) | Propranolol: 4358 (12.88%),  Acetylsalicylic acid: 4037 (11.93%), quetiapine: 2023 (5.98%) | Alprazolam: 2214 (6.54%),  Metoprolol: 1789 (5.29%),  Insulin aspart: 661 (1.95%) |
| FOLIC ACID |  | Methotrexate: 14294 (15.89%),  Capecitabine: 53 (0.06%) | Phenobarbital: 404 (0.45%),  Phenytoin: 252 (0.28%),  Primidone: 213 (0.24%) |
| FUROSEMIDE | Desmopressin: 3 (0.00%) | Acetylsalicylic acid: 20762 (34.11%), losartan: 12333 (20.26%),  Valsartan: 11484 (18.87%) | Metformin: 4902 (8.05%),  Digoxin: 3099 (5.09%),  Propranolol: 1146 (1.88%) |
| GABAPENTIN |  | Baclofen: 6758 (5.40%),  Clonazepam: 5120 (4.09%),  Quetiapine: 4763 (3.80%) | Clonidine: 290 (0.23%),  Ginkgo: 34 (0.03%),  Evening primrose oil: 10 (0.01%) |
| GEMFIBROZIL | Ezetimibe: 35 (0.32%),  Repaglinide: 86 (0.78%),  Simvastatin: 15 (0.14%) | Atorvastatin: 2625 (23.79%),  Rosuvastatin: 493 (4.47%),  Pioglitazone: 136 (1.23%) | Insulin: 501 (4.54%),  Montelukast: 110 (1.00%),  Loperamide: 18 (0.16%) |
| GLIBENCLAMIDE |  | Metformin: 10742 (65.27%),  Acetylsalicylic acid: 4805 (29.20%), hydrochlorothiazide: 1694 (10.29%) | Metoprolol: 2190 (13.31%),  Levothyroxine: 1345 (8.17%),  Empagliflozin: 1270 (7.72%) |
| GLICLAZIDE |  | Acarbose: 2136 (3.17%),  Liraglutide: 254 (0.38%),  Methotrexate: 105 (0.16%) | Empagliflozin: 23656 (35.06%),  Linagliptin: 13096 (19.41%),  Cimetidine: 17 (0.03%) |
| HEMATINIC |  | Magnesium: 499 (3.30%),  Carbamazepine: 55 (0.36%),  Methotrexate: 17 (0.11%) | Zinc: 1089 (7.21%),  Levothyroxine: 402 (2.66%),  Pantoprazole: 372 (2.46%) |
| HYDROCHLOROTHIAZIDE |  | Acetylsalicylic acid: 35357 (34.54%), empagliflozin: 7637 (7.46%),  Sitagliptin: 5513 (5.39%) | Metformin: 24350 (23.78%),  Propranolol: 4183 (4.09%),  Calcium: 3238 (3.16%) |
| HYDROXYCHLOROQUINE | Pimozide: 1 (0.01%) | Methotrexate: 5654 (33.67%),  Gabapentin: 1287 (7.66%),  Azathioprine: 1018 (6.06%) |  |
| HYOSCINE | Potassium citrate: 28 (0.04%)  Potassium chloride: 6 (0.01%), | Dicyclomine: 8625 (11.66%), chlordiazepoxide: 3790 (5.12%),  Adult cold: 3507 (4.74%) |  |
| IBUPROFEN | Ketorolac: 15826 (15.87%) | Dexamethasone: 9771 (9.80%),  Piroxicam: 5335 (5.35%),  Diclofenac: 5000 (5.01%) | Losartan: 3356 (3.37%),  Propranolol: 2072 (2.08%),  Levofloxacin: 1327 (1.33%) |
| INDOMETHACIN | Ketorolac: 2749 (18.40%) | Methylprednisolone: 1431 (9.58%), piroxicam: 1288 (8.62%),  Potassium: 1114 (7.45%) | Losartan: 1036 (6.93%),  Metoprolol: 391 (2.62%),  Propranolol: 327 (2.19%) |
| LANSOPRAZOLE |  | Citalopram: 125 (0.74%),  Methotrexate: 67 (0.40%),  Fluconazole: 52 (0.31%) | Levothyroxine: 655 (3.90%),  Clopidogrel: 417 (2.48%),  Iron: 50 (0.30%) |
| LEVOTHYROXINE |  | Furosemide: 3542 (3.35%),  Midodrine: 33 (0.03%),  Iopromide: 1 (0.00%) | Metformin: 28494 (26.97%),  Pantoprazole: 9923 (9.39%),  Empagliflozin: 9723 (9.20%) |
| LITHIUM |  | Risperidone: 2991 (22.97%),  Olanzapine: 1479 (11.36%),  Sertraline: 1228 (9.43%) | Carbamazepine: 1110 (8.52%),  Clonidine: 120 (0.92%),  Lactulose: 90 (0.69%) |
| LORATADINE |  | Clarithromycin: 188 (0.35%),  Carbamazepine: 58 (0.11%),  Itraconazole: 30 (0.06%) |  |
| LOSARTAN |  | Furosemide: 12333 (5.81%),  Captopril: 2567 (1.21%),  Valsartan: 2047 (0.97%) | Insulin Aspart: 11003 (5.19%),  Insulin: 8061 (3.80%),  Spironolactone: 7031 (3.31%) |
| LOSARTAN POTASSIUM / HYDROCHLOROTHIAZIDE |  | Acetylsalicylic acid: 17765 (35.66%), empagliflozin: 4133 (8.30%),  Sitagliptin: 3263 (6.55%) | Metformin: 13796 (27.69%),  Propranolol: 1942 (3.90%),  Calcium: 1401 (2.81%) |
| MEFENAMIC | Ketorolac: 1774 (11.70%) | Diclofenac: 1012 (6.68%),  Ibuprofen: 705 (4.65%),  Enoxaparin: 554 (3.65%) | Losartan: 481 (3.17%),  Propranolol: 268 (1.77%),  Metoprolol: 204 (1.35%) |
| MELOXICAM | Ketorolac: 7366 (16.06%) | Methylprednisolone: 5776 (12.60%), piroxicam: 2575 (5.62%),  Diclofenac: 2091 (4.56%) | Losartan: 1875 (4.09%),  Metoprolol: 790 (1.72%),  Valsartan: 745 (1.62%) |
| MESALAZINE |  | Azathioprine: 1622 (16.25%),  Sulfasalazine: 128 (1.28%),  Naproxen: 124 (1.24%) | Magnesium hydroxide: 162 (1.62%),  Insulin Aspart: 77 (0.77%),  Insulin: 53 (0.53%) |
| METFORMIN |  | Acetylsalicylic acid: 61401 (27.00%),  Insulin Aspart: 15936 (7.01%),  Insulin: 12857 (5.65%) | Levothyroxine: 28494 (12.53%),  Amlodipine: 28225 (12.41%),  Metoprolol: 27159 (11.94%) |
| METOPROLOL SUCCINATE |  | Sertraline: 932 (3.41%),  Escitalopram: 395 (1.45%),  Diltiazem: 198 (0.72%) | Acetylsalicylic acid: 14045 (51.41%),  Metformin: 4098 (15.00%),  Empagliflozin: 3552 (13.00%) |
| METOPROLOL TARTRATE |  | Acetylsalicylic acid: 12114 (12.87%),  Insulin: 4688 (4.98%),  Hydrochlorothiazide: 4649 (4.94%) | Amlodipine: 5652 (6.01%),  Levothyroxine: 5221 (5.55%),  Metoprolol: 4992 (5.30%) |
| METRONIDAZOLE | Thioridazine: 3 (0.00%) | Ciprofloxacin: 9080 (10.33%),  Ondansetron: 8864 (10.08%),  Famotidine: 7431 (8.45%) | Carbamazepine: 79 (0.09%),  Cholestyramine: 20 (0.02%),  Ergotamine: 14 (0.02%) |
| MONTELUKAST |  | Carbamazepine: 83 (0.07%),  Warfarin: 82 (0.07%),  Olanzapine: 43 (0.03%) | Gemfibrozil: 110 (0.09%) |
| MYCOPHENOLATE MOFETIL |  | Pantoprazole: 1754 (14.95%),  Valganciclovir: 505 (4.30%),  Omeprazole: 409 (3.49%) | Iron: 73 (0.62%),  Fenofibrate: 64 (0.55%) |
| NAPROXEN | Ketorolac: 46201 (30.15%) | Dexamethasone: 19000 (12.40%),  Piroxicam: 12744 (8.32%),  Hydrocortisone: 6491 (4.24%) | Levofloxacin: 3818 (2.49%),  Losartan: 3506 (2.29%),  Propranolol: 1719 (1.12%) |
| NITROGLYCERIN | Sildenafil: 32 (0.04%),  Tadalafil: 31 (0.03%) | Heparin: 14 (0.02%) | Acetylsalicylic acid: 54210 (60.17%),  Acetylcysteine: 1045 (1.16%) |
| NORTRIPTYLINE | Metoclopramide: 144 (0.46%),  Ranolazine: 43 (0.14%),  Linezolid: 8 (0.03%) | Sertraline: 3130 (10.00%),  Acetylsalicylic acid: 3019 (9.65%), escitalopram: 1716 (5.48%) | Warfarin: 76 (0.24%),  Phenytoin: 40 (0.13%),  Cimetidine: 26 (0.08%) |
| OLANZAPINE | Thioridazine: 116 (0.73%),  Pimozide: 74 (0.46%),  Metoclopramide: 26 (0.16%) | Clonazepam: 3129 (19.56%),  Sertraline: 3018 (18.87%),  Biperiden: 2979 (18.62%) | Valproate: 5048 (31.56%),  Clonidine: 285 (1.78%),  Insulin: 90 (0.56%) |
| OMEPRAZOLE |  | Clarithromycin: 1277 (1.80%),  Clopidogrel: 1114 (1.57%),  Citalopram: 770 (1.08%) | Levothyroxine: 3644 (5.13%),  Alprazolam: 3196 (4.50%),  Propranolol: 2536 (3.57%) |
| ONDANSETRON | Fluconazole: 166 (0.10%),  Apomorphine: 7 (0.00%),  Pimozide: 5 (0.00%) | Famotidine: 22501 (14.13%),  Azithromycin: 12454 (7.82%),  Adult cold: 10534 (6.61%) | Cyclophosphamide: 346 (0.22%) |
| PANTOPRAZOLE |  | Mycophenolate mofetil: 1754 (0.73%), methotrexate: 1630 (0.68%),  Fluconazole: 926 (0.39%) | Levothyroxine: 9923 (4.15%),  Warfarin: 3444 (1.44%),  Ferfolic: 1839 (0.77%) |
| PREDNISOLONE | Desmopressin: 81 (0.10%) | Acetylsalicylic acid: 5355 (6.83%), levofloxacin: 3904 (4.98%),  Naproxen: 2366 (3.02%) | Metformin: 2812 (3.59%),  Hydrochlorothiazide: 1439 (1.83%),  Insulin: 1187 (1.51%) |
| PREGABALIN |  | Clonazepam: 1958 (5.10%),  Tizanidine: 1777 (4.63%),  Quetiapine: 1756 (4.57%) |  |
| PROPRANOLOL | Rizatriptan: 243 (0.31%),  Thioridazine: 140 (0.18%) | Escitalopram: 4665 (5.95%),  Fluoxetine: 4358 (5.55%),  Haloperidol: 2610 (3.33%) | Sertraline: 10480 (13.36%),  Acetylsalicylic acid: 10471 (13.35%),  Metformin: 6796 (8.66%) |
| QUETIAPINE | Thioridazine: 218 (0.48%),  Pimozide: 140 (0.31%),  Metoclopramide: 43 (0.09%) | Sertraline: 9650 (21.17%),  Clonazepam: 9169 (20.12%),  Biperiden: 7036 (15.44%) | Insulin: 475 (1.04%),  Clonidine: 469 (1.03%),  Warfarin: 129 (0.28%) |
| RISPERIDONE | Metoclopramide: 7 (0.03%),  Pimozide: 85 (0.31%),  Thioridazine: 226 (0.83%) | Quetiapine: 4880 (17.92%),  Sertraline: 3712 (13.63%),  Lithium: 2991 (10.98%) | Clonidine: 907 (3.33%),  Lamotrigine: 764 (2.81%),  Insulin: 139 (0.51%) |
| ROSUVASTATIN |  | Fenofibrate: 2810 (2.63%),  Warfarin: 899 (0.84%),  Ticagrelor: 579 (0.54%) | Amiodarone: 634 (0.59%) |
| SERTRALINE | Pimozide: 204 (0.29%),  Thioridazine: 149 (0.21%),  Metoclopramide: 83 (0.12%) | Quetiapine: 9650 (13.64%),  Metoprolol: 4070 (5.75%),  risperidone: 3712 (5.25%) | Propranolol: 10480 (14.81%),  Alprazolam: 5658 (8.00%),  Fluphenazine: 159 (0.22%) |
| SIMETHICONE |  |  | Levothyroxine: 1290 (3.19%) |
| SITAGLIPTIN / METFORMIN |  | Acetylsalicylic acid: 12114 (26.13%), hydrochlorothiazide: 4649 (10.03%),  Insulin Aspart: 2735 (5.90%) | Amlodipine: 5652 (12.19%),  Levothyroxine: 5221 (11.26%),  Metoprolol: 4992 (10.77%) |
| SPIRONOLACTONE | Colchicine: 195 (0.53%),  Triamterene: 169 (0.46%),  Potassium citrate: 58 (0.16%) | Acetylsalicylic acid: 14755 (39.77%), captopril: 2375 (6.40%),  Digoxin: 2332 (6.29%) | Valsartan: 7807 (21.04%),  Losartan: 7031 (18.95%),  Metformin: 4909 (13.23%) |
| TAMSULOSIN |  | Celecoxib: 2019 (3.93%),  Prazosin: 1219 (2.37%),  Tadalafil: 1037 (2.02%) | Metoprolol: 3794 (7.38%),  Bisoprolol: 1930 (3.76%),  Carvedilol: 1455 (2.83%) |
| TRIAMTERENE / HYDROCHLOROTHIAZIDE | Spironolactone: 169 (1.12%),  Amiloride: 119 (0.79%),  Eplerenone: 45 (0.30%) | Acetylsalicylic acid: 5224 (34.59%),  Potassium: 4418 (29.26%),  Empagliflozin: 690 (4.57%) | Losartan: 4379 (29.00%),  Valsartan: 2904 (19.23%),  Metformin: 2877 (19.05%) |
| TRIFLUOPERAZINE | Venlafaxine: 428 (2.24%)  Metoclopramide: 174 (0.91%), potassium chloride: 1 (0.01%), potassium citrate: 1 (0.01%), | Clonazepam: 1700 (8.92%),  Alprazolam: 1477 (7.75%),  Quetiapine: 1200 (6.29%) | Metformin: 771 (4.04%),  Trihexyphenidyl: 613 (3.22%),  Insulin: 161 (0.84%) |
| TRIHEXYPHENIDYL | Potassium chloride: 1 (0.02%) | Quetiapine: 1256 (18.87%),  Olanzapine: 589 (8.85%),  Amantadine: 485 (7.29%) | Haloperidol: 942 (14.15%),  Perphenazine: 739 (11.10%),  Trifluoperazine: 613 (9.21%) |
| URSODEOXYCHOLIC ACID |  |  |  |
| VALPROATE |  | Clonazepam: 10960 (19.28%),  Acetylsalicylic acid: 3962 (6.97%),  Carbamazepine: 3858 (6.79%) | Risperidone: 10048 (17.68%),  Olanzapine: 5048 (8.88%),  Nortriptyline: 2667 (4.69%) |
| VALSARTAN | Captopril: 1403 (1.18%),  Enalapril: 196 (0.16%),  Lisinopril: 18 (0.02%) | Furosemide: 11484 (9.66%),  Tacrolimus: 470 (0.40%),  Potassium citrate: 172 (0.14%) | Spironolactone: 7807 (6.57%),  Insulin: 7547 (6.35%),  Eplerenone: 3055 (2.57%) |
| VITAMIN B1 |  |  | Capecitabine: 102 (0.04%),  Fluorouracil: 30 (0.01%) |
| VITAMIN D |  |  | Quetiapine: 37 (0.57%),  Aripiprazole: 7 (0.11%),  Cimetidine: 2 (0.03%) |

This table presents the three most frequent drug interaction partners for each of the 100 most commonly prescribed medications in the IHIO database, categorized by severity level (contraindicated, major, and moderate). Numbers represent the absolute frequency of co-prescription, while percentages (in parentheses) indicate the proportion relative to all prescriptions containing the target drug listed in the first column. Empty cells indicate no interactions were identified in that severity category.
