# Supplementary material for: Prevalence, predictors, and clinical relevance of drug–drug interactions in outpatient prescribing: A national cross-sectional study
Source: PLoS One. 2026 Apr 8;21(4):e0345076. doi: 10.1371/journal.pone.0345076 (PMC13061183; doi:10.1371/journal.pone.0345076)
Supplement: S3 Table — (DOCX) [file pone.0345076.s003.DOCX]

**S3 Table 1.** Distribution of Top Drug-Drug Interactions by Severity Level Across Sex, Age Groups

| ***Classification*** | | | ***Total – top 5 (repetition)*** | | ***Moderate – top 3 (repetition)*** | ***Major – top 3 (repetition)*** | ***Contraindicated – top 3 (repetition)*** |
| --- | --- | --- | --- | --- | --- | --- | --- |
| ***Sex*** | **Male** | ACETYLSALICYLIC ACID – NITROGLYCERIN (28706) | | ACETYLSALICYLIC ACID - NITROGLYCERIN (28706) | | ACETYLSALICYLIC ACID - METFORMIN (27058) | KETOROLAC - NAPROXEN (18048) |
|  |  | ACETYLSALICYLIC ACID – METFORMIN (27058) | | ACETYLSALICYLIC ACID - METOPROLOL TARTRATE (21041) | | ACETYLSALICYLIC ACID - CLOPIDOGREL (24106) | DICLOFENAC - KETOROLAC (8761) |
|  |  | ACETYLSALICYLIC ACID – CLOPIDOGREL (24106) | | ATORVASTATIN - CLOPIDOGREL (18806) | | ACETYLSALICYLIC ACID - HYDROCHLOROTHIAZIDE (13323) | CELECOXIB - KETOROLAC (4426) |
|  |  | ACETYLSALICYLIC ACID - METOPROLOL TARTRATE (21041) | |  | |  |  |
|  |  | ATORVASTATIN – CLOPIDOGREL (18806) | |  | |  |  |
|  | **Female** | ACETYLSALICYLIC ACID – METFORMIN (34343) | | ACETYLSALICYLIC ACID - NITROGLYCERIN (25504) | | ACETYLSALICYLIC ACID - METFORMIN (34343) | KETOROLAC - NAPROXEN (28153) |
|  |  | KETOROLAC – NAPROXEN (28153) | | ACETYLSALICYLIC ACID - METOPROLOL TARTRATE (23625) | | ACETYLSALICYLIC ACID - HYDROCHLOROTHIAZIDE (21620) | DICLOFENAC - KETOROLAC (14250) |
|  |  | ACETYLSALICYLIC ACID – NITROGLYCERIN (25504) | | LEVOTHYROXINE - METFORMIN (21138) | | FAMOTIDINE - ONDANSETRON (15146) | CELECOXIB - KETOROLAC (8894) |
|  |  | ACETYLSALICYLIC ACID - METOPROLOL TARTRATE (23625) | |  | |  |  |
|  |  | ACETYLSALICYLIC ACID – HYDROCHLOROTHIAZIDE (21620) | |  | |  |  |
| ***Age groups*** | **0-18** | CETIRIZINE – DIPHENHYDRAMINE (7190) | | AZITHROMYCIN - THEOPHYLLINE (929) | | CETIRIZINE - DIPHENHYDRAMINE (7190) | KETOROLAC - NAPROXEN (3517) |
|  |  | AZITHROMYCIN – ONDANSETRON (4520) | | RISPERIDONE - VALPROATE (852) | | AZITHROMYCIN - ONDANSETRON (4520) | IBUPROFEN - KETOROLAC (1363) |
|  |  | FAMOTIDINE – ONDANSETRON (3938) | | CLONIDINE - RISPERIDONE (553) | | FAMOTIDINE - ONDANSETRON (3938) | DICLOFENAC - KETOROLAC (1142) |
|  |  | KETOROLAC – NAPROXEN (3517) | |  | |  |  |
|  |  | ADULT COLD – DEXTROMETHORPHAN (3011) | |  | |  |  |
|  | **19-39** | KETOROLAC – NAPROXEN (12548) | | RISPERIDONE - VALPROATE (4185) | | FAMOTIDINE - ONDANSETRON (6522) | KETOROLAC - NAPROXEN (12548) |
|  |  | FAMOTIDINE – ONDANSETRON (6522) | | PROPRANOLOL - SERTRALINE (2772) | | CETIRIZINE - DIPHENHYDRAMINE (6155) | DICLOFENAC - KETOROLAC (4722) |
|  |  | CETIRIZINE – DIPHENHYDRAMINE (6155) | | OLANZAPINE - VALPROATE (1908) | | DEXAMETHASONE - NAPROXEN (5855) | IBUPROFEN - KETOROLAC (2915) |
|  |  | DEXAMETHASONE – NAPROXEN (5855) | |  | |  |  |
|  |  | AZITHROMYCIN – FAMOTIDINE (4896) | |  | |  |  |
|  | **40-64** | ACETYLSALICYLIC ACID – METFORMIN (27972) | | ACETYLSALICYLIC ACID - NITROGLYCERIN (20433) | | ACETYLSALICYLIC ACID - METFORMIN (27972) | KETOROLAC - NAPROXEN (23201) |
|  |  | KETOROLAC – NAPROXEN (23201) | | ACETYLSALICYLIC ACID - METOPROLOL TARTRATE (18505) | | ACETYLSALICYLIC ACID - CLOPIDOGREL (18499) | DICLOFENAC - KETOROLAC (12636) |
|  |  | ACETYLSALICYLIC ACID – NITROGLYCERIN (20433) | | ACETYLSALICYLIC ACID - BISOPROLOL FUMARATE (15852) | | ACETYLSALICYLIC ACID - HYDROCHLOROTHIAZIDE (13016) | CELECOXIB - KETOROLAC (7586) |
|  |  | ACETYLSALICYLIC ACID - METOPROLOL TARTRATE (18505) | |  | |  |  |
|  |  | ACETYLSALICYLIC ACID – CLOPIDOGREL (18499) | |  | |  |  |
|  | **65+** | ACETYLSALICYLIC ACID – NITROGLYCERIN (33350) | | ACETYLSALICYLIC ACID - NITROGLYCERIN (33350) | | ACETYLSALICYLIC ACID - METFORMIN (32926) | KETOROLAC - NAPROXEN (6935) |
|  |  | ACETYLSALICYLIC ACID – METFORMIN (32926) | | ACETYLSALICYLIC ACID - METOPROLOL TARTRATE (25754) | | ACETYLSALICYLIC ACID - HYDROCHLOROTHIAZIDE (21766) | DICLOFENAC - KETOROLAC (4511) |
|  |  | ACETYLSALICYLIC ACID - METOPROLOL TARTRATE (25754) | | ACETYLSALICYLIC ACID - BISOPROLOL FUMARATE (16641) | | ACETYLSALICYLIC ACID - CLOPIDOGREL (18841) | CELECOXIB - KETOROLAC (3420) |
|  |  | ACETYLSALICYLIC ACID – HYDROCHLOROTHIAZIDE (21766) | |  | |  |  |
|  |  | ACETYLSALICYLIC ACID – CLOPIDOGREL (18841) | |  | |  |  |
