# Supplementary material for: Prevalence, predictors, and clinical relevance of drug–drug interactions in outpatient prescribing: A national cross-sectional study
Source: PLoS One. 2026 Apr 8;21(4):e0345076. doi: 10.1371/journal.pone.0345076 (PMC13061183; doi:10.1371/journal.pone.0345076)
Supplement: S4 Table — (DOCX) [file pone.0345076.s004.DOCX]

**S4 Table 1.** Specialty-Specific Drug–Drug Interaction Profiles: Top 5 Interactions Overall and Top 3 Interactions Within Each Severity Category (Major, Moderate, Contraindicated)

| ***specialty*** | ***Total – top 5 (repetition)*** | ***Moderate – top 3 (repetition)*** | ***Major – top 3 (repetition)*** | ***Contraindicated – top 3 (repetition)*** |
| --- | --- | --- | --- | --- |
| ***Aerospace and undersea medicine*** | LEVOFLOXACIN – PREDNISOLONE (14) | LEVOFLOXACIN - NAPROXEN (3) | LEVOFLOXACIN - PREDNISOLONE (14) | KETOROLAC - MELOXICAM (9) |
|  | FAMOTIDINE – LEVOFLOXACIN (9) | ACETAMINOPHEN/CAFFEINE/IBUPROFEN - VALSARTAN (2) | FAMOTIDINE - LEVOFLOXACIN (9) | KETOROLAC - NAPROXEN (8) |
|  | KETOROLAC – MELOXICAM (9) | HEMATINIC - PRIMIDONE (2) | AZITHROMYCIN - FAMOTIDINE (6) |  |
|  | KETOROLAC – NAPROXEN (8) |  |  |  |
|  | AZITHROMYCIN – FAMOTIDINE (6) |  |  |  |
| **Anesthesiology** | PREGABALIN – TIZANIDINE (243) | ACETYLSALICYLIC ACID - NITROGLYCERIN (75) | PREGABALIN - TIZANIDINE (243) | KETOROLAC - MELOXICAM (165) |
|  | BACLOFEN – GABAPENTIN (176) | AMLODIPINE - METFORMIN (69) | BACLOFEN - GABAPENTIN (176) | CELECOXIB - KETOROLAC (36) |
|  | KETOROLAC – MELOXICAM (165) | ACETYLSALICYLIC ACID - METOPROLOL TARTRATE (63) | BACLOFEN - PREGABALIN (150) | KETOROLAC - NAPROXEN (34) |
|  | BACLOFEN – PREGABALIN (150) |  |  |  |
|  | ACETYLSALICYLIC ACID – METFORMIN (121) |  |  |  |
| **Cardiology** | ACETYLSALICYLIC ACID – NITROGLYCERIN (31984) | ACETYLSALICYLIC ACID - NITROGLYCERIN (31984) | ACETYLSALICYLIC ACID - CLOPIDOGREL (27677) | AMLODIPINE / VALSARTAN - CAPTOPRIL (278) |
|  | ACETYLSALICYLIC ACID – CLOPIDOGREL (27677) | ACETYLSALICYLIC ACID - BISOPROLOL FUMARATE (23924) | ACETYLSALICYLIC ACID - HYDROCHLOROTHIAZIDE(15717) | ATORVASTATIN - COLCHICINE (242) |
|  | ACETYLSALICYLIC ACID - BISOPROLOL FUMARATE (23924) | ATORVASTATIN - CLOPIDOGREL (21036) | ACETYLSALICYLIC ACID - FUROSEMIDE (12591) | CAPTOPRIL - VALSARTAN (138) |
|  | ATORVASTATIN – CLOPIDOGREL (21036) |  |  |  |
|  | ACETYLSALICYLIC ACID - METOPROLOL TARTRATE (20434) |  |  |  |
| **Chemotherapy** | CYCLOPHOSPHAMIDE – ONDANSETRON (23) | CYCLOPHOSPHAMIDE - ONDANSETRON (23) | FAMOTIDINE - ONDANSETRON (9) | KETOROLAC - NAPROXEN (1) |
|  | FAMOTIDINE – ONDANSETRON)9( | LEVOTHYROXINE - PANTOPRAZOLE (3) | CHLORPHENIRAMINE - ONDANSETRON (5) |  |
|  | CHLORPHENIRAMINE – ONDANSETRON )5( | ACETYLSALICYLIC ACID - METOPROLOL TARTRATE (2) | GRANISETRON - ONDANSETRON (5) |  |
|  | GRANISETRON – ONDANSETRON (5) |  |  |  |
|  | ONDANSETRON – OXALIPLATIN (5) |  |  |  |
| **Dentistry** | DEXAMETHASONE – IBUPROFEN (230) | ACETYLSALICYLIC ACID - BISOPROLOL FUMARATE (5) | DEXAMETHASONE - IBUPROFEN (230) | IBUPROFEN - KETOROLAC (152) |
|  | IBUPROFEN – KETOROLAC (152) | ACETYLSALICYLIC ACID - METOPROLOL TARTRATE (4) | ACETAMINOPHEN/CAFFEINE/IBUPROFEN - DEXAMETHASONE (59) | ACETAMINOPHEN/CAFFEINE/IBUPROFEN - KETOROLAC (71) |
|  | ACETAMINOPHEN/CAFFEINE/IBUPROFEN – KETOROLAC (71) | ALPRAZOLAM - SERTRALINE (3) | DEXAMETHASONE - MEFENAMIC (54) | KETOROLAC - MEFENAMIC (20) |
|  | ACETAMINOPHEN/CAFFEINE/IBUPROFEN - DEXAMETHASONE (59) |  |  |  |
|  | DEXAMETHASONE - MEFENAMIC (54) |  |  |  |
| **Dermatology** | FOLIC ACID - METHOTREXATE (652) | METFORMIN - PREDNISOLONE (39) | FOLIC ACID - METHOTREXATE (652) | ESCITALOPRAM - FLUCONAZOLE (2) |
|  | FAMOTIDINE - HYDROXYZINE (77) | ACETYLSALICYLIC ACID - METOPROLOL TARTRATE (22) | FAMOTIDINE - HYDROXYZINE (77) | KETOROLAC - NAPROXEN (2) |
|  | CETIRIZINE - HYDROXYZINE (50) | ACETYLSALICYLIC ACID - NITROGLYCERIN (15) | CETIRIZINE - HYDROXYZINE (50) | LINEZOLID - SERTRALINE (2) |
|  | METFORMIN - PREDNISOLONE (39) |  |  |  |
|  | ACETYLSALICYLIC ACID - METFORMIN (35) |  |  |  |
| **Dialysis** | ACETYLSALICYLIC ACID - FUROSEMIDE (3) | ACETYLSALICYLIC ACID - CALCIUM CARBONATE (2) | ACETYLSALICYLIC ACID - FUROSEMIDE (3) | DICLOFENAC - KETOROLAC (1) |
|  | ACETYLSALICYLIC ACID - CALCIUM CARBONATE (2) | ACETYLSALICYLIC ACID - NITROGLYCERIN (2) | FUROSEMIDE - LOSARTAN (2) | POTASSIUM CITRATE - SPIRONOLACTONE (1) |
|  | ACETYLSALICYLIC ACID - NITROGLYCERIN (2) | FERROUS SULFATE - PANTOPRAZOLE (2) | ACETYLSALICYLIC ACID - AMILORIDE (1) |  |
|  | FERROUS SULFATE - PANTOPRAZOLE (2) |  |  |  |
|  | FUROSEMIDE - LOSARTAN (2) |  |  |  |
| **Emergency medicine** | KETOROLAC - NAPROXEN (5228) | ACETYLSALICYLIC ACID - NITROGLYCERIN (995) | CETIRIZINE - DIPHENHYDRAMINE (4732) | KETOROLAC - NAPROXEN (5228) |
|  | CETIRIZINE - DIPHENHYDRAMINE (4732) | ACETYLSALICYLIC ACID - METOPROLOL TARTRATE (993) | AZITHROMYCIN - FAMOTIDINE (4030) | DICLOFENAC - KETOROLAC (4026) |
|  | AZITHROMYCIN - FAMOTIDINE (4030) | AMLODIPINE - METFORMIN (782) | ACETAMINOPHEN / CODEINE - DIPHENHYDRAMINE (3258) | IBUPROFEN - KETOROLAC (2392) |
|  | DICLOFENAC - KETOROLAC (4026) |  |  |  |
|  | ACETAMINOPHEN / CODEINE - DIPHENHYDRAMINE (3258) |  |  |  |
| **Family medicine** | ACETYLSALICYLIC ACID - METFORMIN (135) | AMLODIPINE - METFORMIN (68) | ACETYLSALICYLIC ACID - METFORMIN (135) | KETOROLAC - NAPROXEN (36) |
|  | CETIRIZINE - DIPHENHYDRAMINE (114) | ACETYLSALICYLIC ACID - METOPROLOL TARTRATE (60) | CETIRIZINE - DIPHENHYDRAMINE (114) | DICLOFENAC - KETOROLAC (20) |
|  | AMLODIPINE - METFORMIN (68) | METFORMIN - METOPROLOL TARTRATE (51) | METFORMIN - REPAGLINIDE (49) | IBUPROFEN - KETOROLAC (12) |
|  | ACETYLSALICYLIC ACID - METOPROLOL TARTRATE (60) |  |  |  |
|  | METFORMIN - METOPROLOL TARTRATE (51) |  |  |  |
| **Forensic medicine** | ACETYLSALICYLIC ACID - METFORMIN (51) | AMLODIPINE - METFORMIN (34) | ACETYLSALICYLIC ACID - METFORMIN (51) | KETOROLAC - NAPROXEN (32) |
|  | AMLODIPINE - METFORMIN (34) | ACETYLSALICYLIC ACID - NITROGLYCERIN (30) | ACETYLSALICYLIC ACID - CLOPIDOGREL (21) | ACETAMINOPHEN/CAFFEINE/IBUPROFEN - KETOROLAC (22) |
|  | KETOROLAC - NAPROXEN (32) | ATORVASTATIN - CLOPIDOGREL (26) | ACETYLSALICYLIC ACID - HYDROCHLOROTHIAZIDE (20) | IBUPROFEN - KETOROLAC (13) |
|  | ACETYLSALICYLIC ACID - NITROGLYCERIN (30) |  |  |  |
|  | ATORVASTATIN - CLOPIDOGREL (26) |  |  |  |
| **General practice** | KETOROLAC - NAPROXEN (36507) | ACETYLSALICYLIC ACID - METOPROLOL TARTRATE (12145) | ACETYLSALICYLIC ACID - METFORMIN (21829) | KETOROLAC - NAPROXEN (36507) |
|  | ACETYLSALICYLIC ACID - METFORMIN (21829) | ACETYLSALICYLIC ACID - NITROGLYCERIN (10775) | CETIRIZINE - DIPHENHYDRAMINE (20193) | DICLOFENAC - KETOROLAC (14670) |
|  | CETIRIZINE - DIPHENHYDRAMINE (20193) | AMLODIPINE - METFORMIN (10477) | FAMOTIDINE - ONDANSETRON (17951) | CELECOXIB - KETOROLAC (6531) |
|  | FAMOTIDINE - ONDANSETRON (17951) |  |  |  |
|  | AZITHROMYCIN - FAMOTIDINE (15985) |  |  |  |
| **General surgery** | CIPROFLOXACIN - METRONIDAZOLE (1894) | ACETYLSALICYLIC ACID - NITROGLYCERIN (1316) | CIPROFLOXACIN - METRONIDAZOLE (1894) | DICLOFENAC - KETOROLAC (189) |
|  | ACETYLSALICYLIC ACID - CLOPIDOGREL (1434) | ATORVASTATIN - CLOPIDOGREL (1184) | ACETYLSALICYLIC ACID - CLOPIDOGREL (1434) | KETOROLAC - NAPROXEN (137) |
|  | ACETYLSALICYLIC ACID - NITROGLYCERIN (1316) | ACETYLSALICYLIC ACID - BISOPROLOL FUMARATE (968) | ACETYLSALICYLIC ACID - FUROSEMIDE (1174) | CELECOXIB - KETOROLAC (81) |
|  | ATORVASTATIN - CLOPIDOGREL (1184) |  |  |  |
|  | ACETYLSALICYLIC ACID - FUROSEMIDE (1174) |  |  |  |
| **Genetics** | LEVOTHYROXINE - MAGNESIUM HYDROXIDE (4) | LEVOTHYROXINE - MAGNESIUM HYDROXIDE (4) | ACETAMINOPHEN / CODEINE - ADULT COLD (1) |  |
|  | LEVOTHYROXINE - SIMETHICONE (3) | LEVOTHYROXINE - SIMETHICONE (3) | COLCHICINE - ROSUVASTATIN (1) |  |
|  | ACETAMINOPHEN / CODEINE - ADULT COLD (1) | CALCIUM CARBONATE - LEVOTHYROXINE (1) | INSULIN ASPART - METFORMIN (1) |  |
|  | CALCIUM CARBONATE - LEVOTHYROXINE (1) |  |  |  |
|  | COLCHICINE - ROSUVASTATIN (1) |  |  |  |
| **Geriatrics** | ACETYLSALICYLIC ACID - METFORMIN (43) | ACETYLSALICYLIC ACID - NITROGLYCERIN (22) | ACETYLSALICYLIC ACID - METFORMIN (43) | KETOROLAC - NAPROXEN (16) |
|  | DONEPEZIL - SERTRALINE (38) | METFORMIN - METOPROLOL TARTRATE (21) | DONEPEZIL - SERTRALINE (38) | DICLOFENAC - KETOROLAC (3) |
|  | AZITHROMYCIN - FAMOTIDINE (25) | AMLODIPINE - METFORMIN (18) | AZITHROMYCIN - FAMOTIDINE (25) | CELECOXIB - KETOROLAC (2) |
|  | ACETYLSALICYLIC ACID - SERTRALINE (24) |  |  |  |
|  | QUETIAPINE - SERTRALINE (24) |  |  |  |
| **Hematology and oncology** | ACETYLSALICYLIC ACID - BISOPROLOL FUMARATE (2) | ACETYLSALICYLIC ACID - BISOPROLOL FUMARATE (2) | ACETYLSALICYLIC ACID - ESCITALOPRAM (2) | DICLOFENAC - KETOROLAC (1) |
|  | ACETYLSALICYLIC ACID - ESCITALOPRAM (2) | ACETYLSALICYLIC ACID - LEVOFLOXACIN (1) | ATORVASTATIN - LEVOFLOXACIN (2) | ESCITALOPRAM - METOCLOPRAMIDE (1) |
|  | ATORVASTATIN - LEVOFLOXACIN (2) | ACETYLSALICYLIC ACID - NITROGLYCERIN (1) | CHLORPHENIRAMINE - ONDANSETRON (2) | METOCLOPRAMIDE - QUETIAPINE (1) |
|  | CHLORPHENIRAMINE - ONDANSETRON (2) |  |  |  |
|  | CLONAZEPAM - OXYCODONE (2) |  |  |  |
| **Immunology** | AZITHROMYCIN - FAMOTIDINE (17) | EMPAGLIFLOZIN - GLICLAZIDE (6) | AZITHROMYCIN - FAMOTIDINE (17) |  |
|  | FAMOTIDINE - FORMOTEROL (7) | CARVEDILOL - METFORMIN (4) | FAMOTIDINE - FORMOTEROL (7) |  |
|  | ACETYLSALICYLIC ACID - METFORMIN (6) | CARVEDILOL - TAMSULOSIN (4) | ACETYLSALICYLIC ACID - METFORMIN (6) |  |
|  | EMPAGLIFLOZIN - GLICLAZIDE (6) |  |  |  |
|  | AMLODIPINE - CLOPIDOGREL (4) |  |  |  |
| **Infectious and tropical diseases** | ACETYLSALICYLIC ACID - METFORMIN (599) | ACETYLSALICYLIC ACID - NITROGLYCERIN (367) | ACETYLSALICYLIC ACID - METFORMIN (599) | KETOROLAC - NAPROXEN (404) |
|  | AZITHROMYCIN - FAMOTIDINE (540) | LEVOFLOXACIN - NAPROXEN (363) | AZITHROMYCIN - FAMOTIDINE (540) | DICLOFENAC - KETOROLAC (84) |
|  | FAMOTIDINE - LEVOFLOXACIN (416) | ACETYLSALICYLIC ACID - METOPROLOL TARTRATE (311) | FAMOTIDINE - LEVOFLOXACIN (416) | CELECOXIB - KETOROLAC (65) |
|  | KETOROLAC - NAPROXEN (404) |  |  |  |
|  | ATORVASTATIN - LEVOFLOXACIN (389) |  |  |  |
| **Internal medicine** | ACETYLSALICYLIC ACID - METFORMIN (19477) | EMPAGLIFLOZIN - GLICLAZIDE (14761) | ACETYLSALICYLIC ACID - METFORMIN (19477) | KETOROLAC - NAPROXEN (1135) |
|  | EMPAGLIFLOZIN - GLICLAZIDE (14761) | LEVOTHYROXINE - METFORMIN (13289) | FOLIC ACID - METHOTREXATE (12350) | DICLOFENAC - KETOROLAC (433) |
|  | LEVOTHYROXINE - METFORMIN (13289) | AMLODIPINE - METFORMIN (8974) | INSULIN ASPART - METFORMIN (6434) | CLARITHROMYCIN - DOMPERIDONE (347) |
|  | FOLIC ACID - METHOTREXATE (12350) |  |  |  |
|  | AMLODIPINE - METFORMIN (8974) |  |  |  |
| **Midwifery** | FLUCONAZOLE - METRONIDAZOLE (178) | EMPAGLIFLOZIN - GLICLAZIDE (13) | FLUCONAZOLE - METRONIDAZOLE (178) | ACETYLSALICYLIC ACID - KETOROLAC (5) |
|  | AZITHROMYCIN - METRONIDAZOLE (75) | DICLOFENAC - FLUCONAZOLE (10) | AZITHROMYCIN - METRONIDAZOLE (75) | DICLOFENAC - KETOROLAC (3) |
|  | CIPROFLOXACIN - METRONIDAZOLE (48) | ACETYLSALICYLIC ACID - METOPROLOL TARTRATE (7) | CIPROFLOXACIN - METRONIDAZOLE (48) | KETOROLAC - NAPROXEN (3) |
|  | AZITHROMYCIN - FLUCONAZOLE (35) |  |  |  |
|  | CIPROFLOXACIN - FLUCONAZOLE (24) |  |  |  |
| **Nephrology** | LEVOFLOXACIN - METRONIDAZOLE (4) | LEVOTHYROXINE - METFORMIN (1) | LEVOFLOXACIN - METRONIDAZOLE (4) |  |
|  | CIPROFLOXACIN - SOLIFENACIN (2) |  | CIPROFLOXACIN - SOLIFENACIN (2) |  |
|  | LEVOTHYROXINE - METFORMIN (1) |  |  |  |
| **Neurology** | QUETIAPINE - SERTRALINE (2553) | PROPRANOLOL - SERTRALINE (2215) | QUETIAPINE - SERTRALINE (2553) | KETOROLAC - NAPROXEN (400) |
|  | PROPRANOLOL - SERTRALINE (2215) | ATORVASTATIN - CLOPIDOGREL (1687) | ACETYLSALICYLIC ACID - CLOPIDOGREL (2104) | CELECOXIB - KETOROLAC (376) |
|  | ACETYLSALICYLIC ACID - CLOPIDOGREL (2104) | NORTRIPTYLINE - VALPROATE (1221) | DONEPEZIL - QUETIAPINE (1974) | DICLOFENAC - KETOROLAC (333) |
|  | DONEPEZIL - QUETIAPINE (1974) |  |  |  |
|  | BACLOFEN - GABAPENTIN (1811) |  |  |  |
| **Neurosurgery** | BACLOFEN - GABAPENTIN (2180) | PROPRANOLOL - SERTRALINE (244) |  |  |
|  | CELECOXIB - METHYLPREDNISOLONE (1550) | CIPROFLOXACIN - ZINC (215) | BACLOFEN - GABAPENTIN (2180) | CELECOXIB - KETOROLAC (790) |
|  | ACETAMINOPHEN / CODEINE - GABAPENTIN (858) | CIPROFLOXACIN - DICLOFENAC (213) | CELECOXIB - METHYLPREDNISOLONE (1550) | KETOROLAC - MELOXICAM (682) |
|  | CELECOXIB - KETOROLAC (790) |  | ACETAMINOPHEN / CODEINE - GABAPENTIN (858) | DICLOFENAC - KETOROLAC (371) |
|  | ACETAMINOPHEN / CODEINE - CIPROFLOXACIN (786) |  |  |  |
| **Nutritional sciences** | ACETYLSALICYLIC ACID - METFORMIN (3) | ACETYLSALICYLIC ACID - METOPROLOL TARTRATE (2) | ACETYLSALICYLIC ACID - METFORMIN (3) | DICLOFENAC - KETOROLAC (1) |
|  | ACETAMINOPHEN / CODEINE - ADULT COLD (2) | LEVOTHYROXINE - METFORMIN (2) | ACETAMINOPHEN / CODEINE - ADULT COLD (2) |  |
|  | ACETYLSALICYLIC ACID - MELOXICAM (2) | ACETYLSALICYLIC ACID - BISOPROLOL FUMARATE (1) | ACETYLSALICYLIC ACID - MELOXICAM (2) |  |
|  | ACETYLSALICYLIC ACID - METOPROLOL TARTRATE (2) |  |  |  |
|  | CETIRIZINE - DIPHENHYDRAMINE (2) |  |  |  |
| **Obstetrics and gynecology** | FLUCONAZOLE - METRONIDAZOLE (5207) | HEMATINIC - ZINC (888) | FLUCONAZOLE - METRONIDAZOLE (5207) | DICLOFENAC - KETOROLAC (49) |
|  | AZITHROMYCIN - METRONIDAZOLE (3312) | METFORMIN - SPIRONOLACTONE (462) | AZITHROMYCIN - METRONIDAZOLE (3312) | KETOROLAC - MEFENAMIC (19) |
|  | AZITHROMYCIN - FLUCONAZOLE (1921) | LEVOTHYROXINE - METFORMIN (267) | AZITHROMYCIN - FLUCONAZOLE (1921) | KETOROLAC - NAPROXEN (16) |
|  | DICLOFENAC - ENOXAPARIN (1650) |  |  |  |
|  | CIPROFLOXACIN - METRONIDAZOLE (1110) |  |  |  |
| **Occupational medicine** | ESCITALOPRAM - TRAZODONE (22) | ACETYLSALICYLIC ACID - METOPROLOL TARTRATE (11) | ESCITALOPRAM - TRAZODONE (22) | IBUPROFEN - KETOROLAC (16) |
|  | ACETYLSALICYLIC ACID - METFORMIN (19) | AMLODIPINE - METFORMIN (9) | ACETYLSALICYLIC ACID - METFORMIN (19) | KETOROLAC - NAPROXEN (6) |
|  | IBUPROFEN - KETOROLAC (16) | METFORMIN - METOPROLOL TARTRATE (9) | ACETYLSALICYLIC ACID - HYDROCHLOROTHIAZIDE (11) | KETOROLAC - MELOXICAM (3) |
|  | ACETYLSALICYLIC ACID - HYDROCHLOROTHIAZIDE (11) |  |  |  |
|  | ACETYLSALICYLIC ACID - METOPROLOL TARTRATE (11) |  |  |  |
| **Ophthalmology** | FOLIC ACID - METHOTREXATE (107) | ACETYLSALICYLIC ACID - NITROGLYCERIN (33) | FOLIC ACID - METHOTREXATE (107) | SERTRALINE - THIORIDAZINE (6) |
|  | ACETYLSALICYLIC ACID - METFORMIN (40) | AMLODIPINE - METFORMIN (33) | ACETYLSALICYLIC ACID - METFORMIN (40) | IBUPROFEN - KETOROLAC (4) |
|  | CIPROFLOXACIN - PREDNISOLONE (34) | ACETYLSALICYLIC ACID - METOPROLOL TARTRATE (31) | CIPROFLOXACIN - PREDNISOLONE (34) | CAPTOPRIL - VALSARTAN (2) |
|  | ACETYLSALICYLIC ACID - NITROGLYCERIN (33) |  |  |  |
|  | AMLODIPINE - METFORMIN (33) |  |  |  |
| **Oral and maxillofacial disorders** | DEXAMETHASONE - IBUPROFEN (4) | KETOROLAC - LOSARTAN (1) | DEXAMETHASONE - IBUPROFEN (4) |  |
|  | CLONAZEPAM - METHOCARBAMOL (1) |  | CLONAZEPAM - METHOCARBAMOL (1) |  |
|  | CLONAZEPAM - QUETIAPINE (1) |  | CLONAZEPAM - QUETIAPINE (1) |  |
|  | KETOROLAC - LOSARTAN (1) |  |  |  |
| **Oral and maxillofacial surgery** | DEXAMETHASONE - IBUPROFEN (67) |  |  |  |
|  | IBUPROFEN - KETOROLAC (56) | ACETYLSALICYLIC ACID - METOPROLOL TARTRATE (2) | DEXAMETHASONE - IBUPROFEN (67) | IBUPROFEN - KETOROLAC (56) |
|  | ACETAMINOPHEN / CODEINE - DEXAMETHASONE (5) | METFORMIN - METOPROLOL TARTRATE (2) | ACETAMINOPHEN / CODEINE - DEXAMETHASONE (5) | ACETAMINOPHEN/CAFFEINE/IBUPROFEN - KETOROLAC (5) |
|  | ACETAMINOPHEN/CAFFEINE/IBUPROFEN - KETOROLAC (5) | ACETYLSALICYLIC ACID - CALCIUM CARBONATE (1) | DICLOFENAC - IBUPROFEN (5) | KETOROLAC - NAPROXEN (1) |
|  | DICLOFENAC - IBUPROFEN (5) |  |  |  |
| **Orthopedics** | CELECOXIB - KETOROLAC (3632) | CIPROFLOXACIN - DICLOFENAC (312) | MELOXICAM - METHYLPREDNISOLONE (3145) | CELECOXIB - KETOROLAC (3632) |
|  | MELOXICAM - METHYLPREDNISOLONE (3145) | CALCIUM+VITAMIN D3 - CIPROFLOXACIN (244) | CELECOXIB - METHYLPREDNISOLONE (2705) | KETOROLAC - MELOXICAM (2543) |
|  | CELECOXIB - METHYLPREDNISOLONE (2705) | ACETYLSALICYLIC ACID - CALCIUM+VITAMIN D3 (219) | DICLOFENAC - METHYLPREDNISOLONE (1755) | DICLOFENAC - KETOROLAC (2220) |
|  | KETOROLAC - MELOXICAM (2543) |  |  |  |
|  | DICLOFENAC - KETOROLAC (2220) |  |  |  |
| **Other** | ACETYLSALICYLIC ACID - NITROGLYCERIN (974) | ACETYLSALICYLIC ACID - NITROGLYCERIN (974) | ACETYLSALICYLIC ACID - METFORMIN (732) | DICLOFENAC - KETOROLAC (24) |
|  | ACETYLSALICYLIC ACID - METOPROLOL TARTRATE (740) | ACETYLSALICYLIC ACID - METOPROLOL TARTRATE (740) | ACETYLSALICYLIC ACID - HYDROCHLOROTHIAZIDE (503) | KETOROLAC - NAPROXEN (19) |
|  | ACETYLSALICYLIC ACID - METFORMIN (732) | AMLODIPINE - METFORMIN (357) | CIPROFLOXACIN - METRONIDAZOLE (421) | CELECOXIB - KETOROLAC (15) |
|  | ACETYLSALICYLIC ACID - HYDROCHLOROTHIAZIDE (503) |  |  |  |
|  | CIPROFLOXACIN - METRONIDAZOLE (421) |  |  |  |
| **Otorhinolaryngology** | ONDANSETRON - PROMETHAZINE (95) | HYDROCHLOROTHIAZIDE - PROPRANOLOL (62) | ONDANSETRON - PROMETHAZINE (95) | KETOROLAC - NAPROXEN (17) |
|  | ACETYLSALICYLIC ACID - METFORMIN (66) | ACETYLSALICYLIC ACID - METOPROLOL TARTRATE (57) | ACETYLSALICYLIC ACID - METFORMIN (66) | DICLOFENAC - KETOROLAC (10) |
|  | HYDROCHLOROTHIAZIDE - PROPRANOLOL (62) | METFORMIN - METOPROLOL TARTRATE (50) | ACETAMINOPHEN / CODEINE - ADULT COLD (55) | DOMPERIDONE - FLUCONAZOLE (9) |
|  | ACETYLSALICYLIC ACID - METOPROLOL TARTRATE (57) |  |  |  |
|  | ACETAMINOPHEN / CODEINE - ADULT COLD (55) |  |  |  |
| **PT and rehabilitation** | METHYLPREDNISOLONE - NAPROXEN (388) | ALPRAZOLAM - FLUOXETINE (51) | METHYLPREDNISOLONE - NAPROXEN (388) | CELECOXIB - KETOROLAC (139) |
|  | GABAPENTIN - TIZANIDINE (222) | ACETYLSALICYLIC ACID - METOPROLOL TARTRATE (42) | GABAPENTIN - TIZANIDINE (222) | DICLOFENAC - KETOROLAC (107) |
|  | MELOXICAM - METHYLPREDNISOLONE (150) | ACETYLSALICYLIC ACID - NITROGLYCERIN (33) | MELOXICAM - METHYLPREDNISOLONE (150) | KETOROLAC - MELOXICAM (94) |
|  | BACLOFEN - GABAPENTIN (147) |  |  |  |
|  | PREGABALIN - TIZANIDINE (144) |  |  |  |
| **Pediatrics** | FOLIC ACID - METHOTREXATE (417) | LEVOTHYROXINE - METFORMIN (194) | FOLIC ACID - METHOTREXATE (417) | KETOROLAC - NAPROXEN (83) |
|  | ACETYLSALICYLIC ACID - METFORMIN (316) | ACETYLSALICYLIC ACID - METOPROLOL TARTRATE (180) | ACETYLSALICYLIC ACID - METFORMIN (316) | IBUPROFEN - KETOROLAC (35) |
|  | AZITHROMYCIN - ONDANSETRON (273) | RISPERIDONE - VALPROATE (174) | AZITHROMYCIN - ONDANSETRON (273) | DICLOFENAC - KETOROLAC (28) |
|  | CAPTOPRIL - FUROSEMIDE (241) |  |  |  |
|  | FAMOTIDINE - ONDANSETRON (232) |  |  |  |
| **Psychiatry** | CLONAZEPAM - VALPROATE (8058) | RISPERIDONE - VALPROATE (8019) | CLONAZEPAM - VALPROATE (8058) | TRIFLUOPERAZINE - VENLAFAXINE (265) |
|  | RISPERIDONE - VALPROATE (8019) | PROPRANOLOL - SERTRALINE (5110) | CLONAZEPAM - QUETIAPINE (6346) | RISPERIDONE - THIORIDAZINE (170) |
|  | CLONAZEPAM - QUETIAPINE (6346) | OLANZAPINE - VALPROATE (3810) | BIPERIDEN - QUETIAPINE (5786) | PIMOZIDE - SERTRALINE (166) |
|  | BIPERIDEN - QUETIAPINE (5786) |  |  |  |
|  | PROPRANOLOL - SERTRALINE (5110) |  |  |  |
| **Radiation oncology** | GRANISETRON - ONDANSETRON (205) | CYCLOPHOSPHAMIDE - ONDANSETRON (152) | GRANISETRON - ONDANSETRON (205) | KETOROLAC - NAPROXEN (27) |
|  | CAPECITABINE - PANTOPRAZOLE (172) | DEXAMETHASONE - METFORMIN (52) | CAPECITABINE - PANTOPRAZOLE (172) | FLUCONAZOLE - ONDANSETRON (14) |
|  | ONDANSETRON - OXALIPLATIN (157) | ACETYLSALICYLIC ACID - METOPROLOL TARTRATE (39) | ONDANSETRON - OXALIPLATIN (157) | DICLOFENAC - KETOROLAC (12) |
|  | CYCLOPHOSPHAMIDE - ONDANSETRON (152) |  |  |  |
|  | CHLORPHENIRAMINE - ONDANSETRON (120) |  |  |  |
| **Radiology** | ACETYLSALICYLIC ACID - CAPTOPRIL (1) | ACETYLSALICYLIC ACID - METOPROLOL SUCCINATE (1) | ACETYLSALICYLIC ACID - CAPTOPRIL (1) |  |
|  | ACETYLSALICYLIC ACID - METOPROLOL SUCCINATE (1) | DICLOFENAC - LOSARTAN (1) | ADULT COLD - AMANTADINE (1) |  |
|  | ADULT COLD - AMANTADINE (1) | DICLOFENAC - METOPROLOL (1) | ADULT COLD - SERTRALINE (1) |  |
|  | ADULT COLD - SERTRALINE (1) |  |  |  |
|  | APIXABAN - DICLOFENAC (1) |  |  |  |
| **Social medicine** | ACETYLSALICYLIC ACID - HYDROCHLOROTHIAZIDE (108) | ACETYLSALICYLIC ACID - METOPROLOL TARTRATE (63) | ACETYLSALICYLIC ACID - HYDROCHLOROTHIAZIDE (108) | KETOROLAC - NAPROXEN (4) |
|  | ACETYLSALICYLIC ACID - METOPROLOL TARTRATE (63) | AMLODIPINE - METFORMIN (52) | ACETYLSALICYLIC ACID - METFORMIN (61) | AMLODIPINE / VALSARTAN - ENALAPRIL (2) |
|  | ACETYLSALICYLIC ACID - METFORMIN (61) | HYDROCHLOROTHIAZIDE - METFORMIN (50) | ACETYLSALICYLIC ACID - INDAPAMIDE (19) | DICLOFENAC - KETOROLAC (2) |
|  | AMLODIPINE - METFORMIN (52) |  |  |  |
|  | HYDROCHLOROTHIAZIDE - METFORMIN (50) |  |  |  |
| **Sports medicine** | ACETYLSALICYLIC ACID - METFORMIN (41) | ACETYLSALICYLIC ACID - METOPROLOL TARTRATE (24) | ACETYLSALICYLIC ACID - METFORMIN (41) | KETOROLAC - NAPROXEN (10) |
|  | DULOXETINE - NAPROXEN (29) | ACETYLSALICYLIC ACID - NITROGLYCERIN (19) | DULOXETINE - NAPROXEN (29) | DICLOFENAC - KETOROLAC (8) |
|  | CELECOXIB - DULOXETINE (27) | METFORMIN - METOPROLOL TARTRATE (17) | CELECOXIB - DULOXETINE (27) | KETOROLAC - MELOXICAM (6) |
|  | ACETYLSALICYLIC ACID - METOPROLOL TARTRATE (24) |  |  |  |
|  | ACETYLSALICYLIC ACID - HYDROCHLOROTHIAZIDE (22) |  |  |  |
| **Traditional Iranian medicine** | ACETYLSALICYLIC ACID - METFORMIN (54) | ACETYLSALICYLIC ACID - NITROGLYCERIN (46) | ACETYLSALICYLIC ACID - METFORMIN (54) | DICLOFENAC - KETOROLAC (14) |
|  | ACETYLSALICYLIC ACID - NITROGLYCERIN (46) | ACETYLSALICYLIC ACID - METOPROLOL TARTRATE (41) | FAMOTIDINE - ONDANSETRON (41) | IBUPROFEN - KETOROLAC (3) |
|  | ACETYLSALICYLIC ACID - METOPROLOL TARTRATE (41) | AMLODIPINE - METFORMIN (29) | ACETYLSALICYLIC ACID - HYDROCHLOROTHIAZIDE (36) | CAPTOPRIL - VALSARTAN (2) |
|  | FAMOTIDINE - ONDANSETRON (41) |  |  |  |
|  | ACETYLSALICYLIC ACID - HYDROCHLOROTHIAZIDE (36) |  |  |  |
| **Urology** | CIPROFLOXACIN - DICLOFENAC (1208) | CIPROFLOXACIN - DICLOFENAC (1208) | CELECOXIB - TAMSULOSIN (932) | DICLOFENAC - KETOROLAC (308) |
|  | CELECOXIB - TAMSULOSIN (932) | CELECOXIB - OFLOXACIN (328) | TADALAFIL - TAMSULOSIN (861) | KETOROLAC - NAPROXEN (127) |
|  | TADALAFIL - TAMSULOSIN (861) | CELECOXIB – LEVOFLOXACIN (240) | DICLOFENAC - HYDROCHLOROTHIAZIDE (591) | CELECOXIB - KETOROLAC (84) |
|  | DICLOFENAC - HYDROCHLOROTHIAZIDE (591) |  |  |  |
|  | PRAZOSIN - TAMSULOSIN (575) |  |  |  |
